# A Genome-Wide Linkage Study for Chronic Obstructive Pulmonary Disease in a Dutch Genetic Isolate Identifies Novel Rare Candidate Variants

**DOI:** 10.3389/fgene.2018.00133

**Published:** 2018-04-19

**Authors:** Ivana Nedeljkovic, Natalie Terzikhan, Judith M. Vonk, Diana A. van der Plaat, Lies Lahousse, Cleo C. van Diemen, Brian D. Hobbs, Dandi Qiao, Michael H. Cho, Guy G. Brusselle, Dirkje S. Postma, H. M. Boezen, Cornelia M. van Duijn, Najaf Amin

**Affiliations:** ^1^Department of Epidemiology, Erasmus Medical Center, Rotterdam, Netherlands; ^2^Department of Respiratory Medicine, Ghent University Hospital, Ghent, Belgium; ^3^Department of Epidemiology, University Medical Center Groningen, University of Groningen, Groningen, Netherlands; ^4^Groningen Research Institute for Asthma and COPD, University Medical Center Groningen, University of Groningen, Groningen, Netherlands; ^5^Pharmaceutical Care Unit, Department of Bioanalysis, Ghent University, Ghent, Belgium; ^6^Department of Genetics, University Medical Center Groningen, University of Groningen, Groningen, Netherlands; ^7^Channing Division of Network Medicine, Brigham and Women’s Hospital, Boston, MA, United States; ^8^Division of Pulmonary and Critical Care Medicine, Brigham and Women’s Hospital, Boston, MA, United States; ^9^Department of Respiratory Medicine, Erasmus Medical Center, Rotterdam, Netherlands; ^10^Department of Pulmonary Medicine and Tuberculosis, University Medical Center Groningen, University of Groningen, Groningen, Netherlands

**Keywords:** COPD, genetic linkage analysis, genetic isolate, rare variants, chromosome 11

## Abstract

Chronic obstructive pulmonary disease (COPD) is a complex and heritable disease, associated with multiple genetic variants. Specific familial types of COPD may be explained by rare variants, which have not been widely studied. We aimed to discover rare genetic variants underlying COPD through a genome-wide linkage scan. Affected-only analysis was performed using the 6K Illumina Linkage IV Panel in 142 cases clustered in 27 families from a genetic isolate, the Erasmus Rucphen Family (ERF) study. Potential causal variants were identified by searching for shared rare variants in the exome-sequence data of the affected members of the families contributing most to the linkage peak. The identified rare variants were then tested for association with COPD in a large meta-analysis of several cohorts. Significant evidence for linkage was observed on chromosomes 15q14–15q25 [logarithm of the odds (LOD) score = 5.52], 11p15.4–11q14.1 (LOD = 3.71) and 5q14.3–5q33.2 (LOD = 3.49). In the chromosome 15 peak, that harbors the known COPD locus for nicotinic receptors, and in the chromosome 5 peak we could not identify shared variants. In the chromosome 11 locus, we identified four rare (minor allele frequency (MAF) <0.02), predicted pathogenic, missense variants. These were shared among the affected family members. The identified variants localize to genes including neuroblast differentiation-associated protein (*AHNAK*), previously associated with blood biomarkers in COPD, phospholipase C Beta 3 (*PLCB3*), shown to increase airway hyper-responsiveness, solute carrier family 22-A11 (*SLC22A11*), involved in amino acid metabolism and ion transport, and metallothionein-like protein 5 (*MTL5*), involved in nicotinate and nicotinamide metabolism. Association of *SLC22A11* and *MTL5* variants were confirmed in the meta-analysis of 9,888 cases and 27,060 controls. In conclusion, we have identified novel rare variants in plausible genes related to COPD. Further studies utilizing large sample whole-genome sequencing should further confirm the associations at chromosome 11 and investigate the chromosome 15 and 5 linked regions.

## Introduction

Chronic obstructive pulmonary disease is a common and complex disease, and one of the leading causes of death worldwide (Lozano and Naghavi, 2012). Previous studies provided heritability estimates for COPD of 20% to even 60% (Ingebrigtsen et al., 2010; Zhou et al., 2013). Both rare variants with a large impact and common variants with a modest impact on the risk to develop COPD have been identified. The *SERPINA1* gene at chromosome 14q32.13, encoding AAT, was in fact the first gene identified to be associated with COPD (Laurell and Eriksson, 1963; Bashir et al., 2016). Rare variants in *SERPINA1* are known to contribute to COPD risk in AAT deficiency in homozygous and heterozygous carriers of the low-frequency Z allele (Foreman et al., 2017). In an exome study of severe, early-onset families, Qiao et al. (2016) identified several genes with rare variants segregating in at least two pedigrees. In extended families, genetic linkage studies have found evidence of linkage to chromosomes 2q, 6q, 8p, 12p, and 19q, among others (Silverman et al., 2002; Palmer et al., 2003). However, many initially promising findings from linkage or exome sequencing in candidate-gene studies could not be replicated in subsequent analyses (Hersh et al., 2005).

Common variants in several genes have been identified in multiple GWAS, to be associated with COPD or obstructive lung function impairment. Among consistently replicated loci in GWAS are genes on chromosome 4 – Hedgehog-interacting protein (*HHIP*) and Family with sequence similarity 13 member A (*FAM13A*), chromosome 5 – 5-hydroxytryptamine receptor 4 (*HTR4*), chromosome 15 – Nicotinic cholinergic receptors (*CHRNA3/5*) and Ion-responsive element binding protein 2 (*IREB2*) and chromosome 19 – Cytochrome P450 family gene (*CYP2A6*), member RAS oncogene family gene (*RAB4B*) and Egl-9 family hypoxic-inducible factor 2 (*EGLN2*) (Hobbs et al., 2017; Wain et al., 2017). However, only few loci identified in GWAS could be functionally explained.

Despite the undeniable progress in understanding the genetic origins of COPD, a major part of its heritability remains unexplained. A complicating factor in studies on the genetics of COPD is that COPD is considered a complex genetic trait, i.e., multiple, possibly interacting, genetic and environmental factors are involved. Therefore, there is a need for fine mapping techniques that can identify functional, rare variants with large effects explaining specific types of COPD. Rare variant association studies can be carried out in relatively small sample sizes when using family-based settings (Auer and Lettre, 2015). In a genetically isolated population, alleles that are found at low or very-low (rare) frequencies in control samples may reach much higher proportions due to a limited number of founder individuals, genetic drift, minimal immigration, and high inbreeding (Pardo et al., 2005). Therefore, attempting to identify risk genes for COPD in populations that are relatively genetically and environmentally homogeneous could be beneficial (Van Diemen et al., 2010).

This study uses the ERF study, a Dutch genetically isolated population, to localize and identify rare genetic variants and subsequently shows the relevance of these variants in the general population by performing an association analysis in a large sample.

## Materials and Methods

### Study Populations

#### Linkage Study

The linkage study was performed in 142 related participants from the ERF study. ERF is a family-based cohort study, studied as part of the Genetic Research in Isolated Population (GRIP) program. It is based in a genetically isolated community from the south-west area of the Netherlands, set up to investigate genes underlying different quantitative traits and common diseases (Pardo et al., 2005). The participants of ERF are living descendants of 22 couples from the religious isolate in the 19th century, who had at least six children baptized in the community church. The baseline data collection for over 3,000 people was conducted between June 2002 and February 2005. These individuals are related to each other through multiple lines of descent in a single large pedigree spanning 23 generations and connecting over 23,000 individuals. In 2015 a follow-up data collection for 1,500 participants was performed by reviewing general practitioner’s records, including letters from the specialists and spirometry reports and medication use. In total 192 probable COPD cases were identified in the follow-up. The COPD diagnosis was confirmed by respiratory specialists based on an obstructive lung function, i.e., the ratio of Forced Expiratory Volume in one second over the Forced Vital Capacity (FEV_1_/FVC) < 0.7, with or without medication use (*n* = 116). If the information on FVC was missing (*n* = 14), the following criteria for COPD were used: FEV_1_ < 80%, use of respiratory medication and a COPD diagnosis in the report of the respiratory specialist to the general practitioner. If no lung function measurement was available (*n* = 15), COPD diagnosis was based on: medication use with CT-scan of the lungs indicating COPD and/or a history of frequent COPD exacerbations mentioned in the medical documents. Thus, the COPD diagnosis could be confirmed for 145 participants, of which 3 did not have genotyping data, resulting in the final sample size for the linkage study of 142 COPD cases.

#### Association Study

The association analysis was performed using data from the RS (1,588 cases and 9,784 controls), the LLS (1,647 cases and 9,530 controls), the VlaVla study (375 cases and 1,019 controls) and the data from the study of Hobbs et al. (2016) (6,161 cases and 6,004 controls), in addition to the ERF study (117 cases and 1,091 controls).

Rotterdam Study is a prospective, population-based study (Ikram et al., 2017), focusing on the diseases in the participants aged 45 or older. The COPD diagnosis in the RS was defined as having pre-bronchodilator obstructive spirometry (FEV_1_/FVC < 0.7), assessed either by spirometry in the research center or by reviewing medical histories of the participants. Spirometry was performed by trained paramedical personnel, according to the guidelines of the American Thoracic Society/European Respiratory Society (ATS/ERS). In absence of interpretable spirometry measures, all medical information of subjects regularly using respiratory medication was reviewed, including files from specialists and general practitioners, to confirm a diagnosis of COPD. Both ERF and RS have been approved by the Medical Ethics Committee of the Erasmus Medical Center. All participants provided written informed consent to participate in the study and to obtain information from their treating physicians.

Lifelines study is a multi-disciplinary prospective population-based cohort of the Northern provinces of the Netherlands with a three generation design, focusing on the onset of common complex diseases (Scholtens et al., 2015). COPD was defined as having pre-bronchodilator FEV1/FVC < 0.7, assessed by spirometry using a Welch Allyn Version 1.6.0.489, PC-based SpiroPerfect with Ca Workstation software. All subjects provided written informed consent and the study was approved by the Medical Ethics Committee of the University Medical Center Groningen, Groningen, Netherlands.

The VlaVla is a prospective, Dutch population-based cohort including individuals from Vlagtwedde (a rural area) and Vlaardingen (an urban area), aimed to gain insight into the risk factors for chronic airway diseases and lung function (Van Diemen et al., 2005). COPD was defined as having pre-bronchodilator FEV_1_/FVC < 0.7. Data of the last survey in 1989/1990 were used and spirometry data were collected by performing a slow inspiratory maneuver, using a water-sealed spirometer (Lode instruments, Groningen, Netherlands). The Committee on Human Subjects in Research of the University of Groningen reviewed the study and affirmed the safety of the protocol and study design and all participants gave their written informed consent.

In the study by Hobbs et al. (2016) COPD cases were defined as having FEV1/FVC ≤ 0.7 and FEV1 ≤ 80% of the predicted value. It was multi-ethnic study with Asian, African, and European ancestry individuals. Institutional review board approval and written informed consent were obtained for all these cohorts. For more details please refer to their publication (Hobbs et al., 2016).

### Genotyping

#### DNA Isolation

For all participants, DNA was extracted from venous blood using the salting out method (Miller et al., 1988).

#### Linkage Array

For the linkage analysis genotyping was performed using the 6K Illumina Linkage IV panel (Illumina, San Diego, CA, United States). Further, QC was performed involving exclusion of the variants with call rate <98%, those diverging from Hardy–Weinberg equilibrium (*P <* 10^-8^) and X-chromosome variants and participants with an overall call rate <96%. Mendelian inconsistencies were designated as missing genotypes. The final dataset comprised 5,250 autosomal SNVs in 3,018 participants.

#### Exome-Sequencing and Genotyping

The sequencing and genotyping in the ERF study have been described elsewhere (Amin et al., 2017). In short, for 1,336 ERF participants whole exome sequencing was performed at a mean depth of 74× (Agilent, v4 capture). After QC, 543,954 SNVs in 1,327 participants were retained. For 1,527 individuals whose exomes were not sequenced, the Illumina Infinium HumanExome BeadChip v1.1 was used for genotyping and variant calling was done using Genome Studio. After QC 70,000 polymorphic SNVs in 1,515 participants were retrieved. Of these, the overlap with COPD status information was available for 636 participants (59 cases and 577 controls) with exome-sequence and 572 participants (58 cases and 514 controls) with exome-chip data. The cases overlap with the sample used in the linkage analysis. The ERF data is available in the EGA public repository^1^ with ID number: EGAS00001001134.

The RS was genotyped using Illumina 550K and Illumina 610K and 660K arrays, and genotyping QC was done as described elsewhere (Iglesias et al., 2017). HRC imputation panel (McCarthy et al., 2015) was used for imputation. File preparation and imputation was done as described elsewhere (Iglesias et al., 2017). In the final dataset we included 11,372 participants of RS (cases and controls) with HRC imputed genotype data available.

In LLS and VlaVla the genotyping was done using Illumina CytoSNP-12 arrays and QC was done as described elsewhere (De Jong et al., 2015). The GoNL panel was used for imputation of LLS and VlaVla and was done as described elsewhere (Scholtens et al., 2015). The final dataset included 11,177 participants of LLS and 1,394 of VlaVla.

In Hobbs et al. (2016) work all individuals were genotyped using the Illumina HumanExome arrays (v1.1 and v1.2; Illumina, San Diego, CA, United States). For more information please refer to their publication (Hobbs et al., 2016).

### Statistical Analyses

#### Genome-Wide Linkage Analysis

For the genome-wide linkage analysis, 142 related COPD cases from ERF were used. The cases were linked in a single large pedigree of 23 generations. However, due to the linkage software restraints, the cases were clustered into 27 smaller (≤24 bits) families using PEDCUT software (Liu et al., 2008). We used HaploPainter (Thiele and Nürnberg, 2005) to illustrate all 27 pedigrees (Supplementary Figure S1). We then performed affected-only parametric linkage analysis in MERLIN software (Abecasis et al., 2002) using incomplete penetrance and no phenocopies for both dominant (0, 0.5, 0.5) and recessive models (0, 0, 0.5) (Durner et al., 1999). The measure of the likelihood of linkage is the LOD score and we considered LOD ≥ 3.3 to be statistically significant. Further we performed per-family analysis for significant regions to identify the families with COPD cases contributing the most to the LOD score.

#### Identification of Variants in the Identified Regions

Next, we used exome-sequence data in ERF to identify rare variants that may explain the identified linkage peaks. For this, among all variants in this region we selected only variants with predicted damaging effects on protein (missense and stop-coding) based on the FunctionGVS column of the SeattleSeq Annotation database^2^ from the National Heart, Lung and Blood Institute (NHLBI) and with MAF < 0.05 in the general population (1000 Genomes). As frequencies in a genetically isolated population may be inflated or deflated due to genetic drift (Pardo et al., 2005), we used the MAF from the general population for filtering. We selected variants shared among most (>50%) of the affected family members as candidate variants.

A formal test of association was performed for the identified candidate variants in each study – ERF, in samples with exome-sequence (*N* = 636) and in exome-chip (*N* = 572) data, in three RS cohorts (RS-I, RS-II, and RS-III), using the HRC imputed data (*N* = 11,372), the LLS (*N* = 11,177), the VlaVla cohort (*N* = 1,394) and the Hobbs et al. (2016) results (*N* = 11,797). For this analysis, in ERF we used “*seqMeta*” package in R (Voorman et al., 2013) to perform single-variant analysis, adjusted for age, sex, and smoking status (current/past/never smoking). Logistic regression analysis was used to associate the variants in the RS and the VlaVla cohort, using SPSS software (Norušis, 1992) and in LLS, using PLINK (Purcell et al., 2007), applying the same models as used in ERF. Variants were excluded from the analysis if the minor allele count was less than five in either the case or the control category. Summary statistics for identified the variants were extracted from the results of Hobbs et al. (2016). A fixed-effects meta-analysis was performed with the summary statistics from all studies using the “*rmeta*” package in R (Lumley, 2011).

#### Functional Look-Up of the Genes

We investigated the Ingenuity Knowledge Base for functional annotation and look up of the genes, harboring the identified variants (IPA, Qiagen bioinformatics) (IPA, 2015). Furthermore, we consulted the Gene network tool (Fehrmann et al., 2015), a bioinformatics database containing co-expression data, functional predictions from gene ontology, Biocarta and the KEGG to investigate our findings.

## Results

**FIGURE 1:**
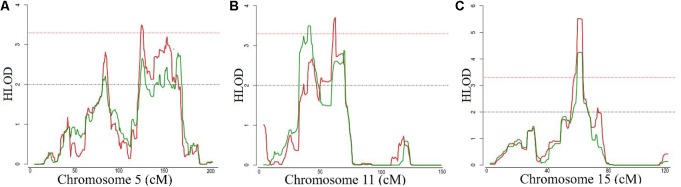
Logarithm of the odds (LOD) score plot for the regions at **(A)** chromosomes 5, **(B)** 11 and **(C)** 15. *X*-axis shows the chromosomal position in cM and the *Y*-axis shows the heterogeneity log of odds score (HLOD) score. Red line represents HLOD scores for recessive and green line for dominant model. Dashed red line represents the level of significance (HLOD = 3.3), while dashed black line represents the suggestive level (HLOD = 2).

The general characteristics of the study samples are presented in **Table 1**. All 27 families included in the linkage analyses in ERF are depicted in Supplementary Figure S1. The affected relatives were mainly smokers: 81.7% of the cases included in the linkage analyses were current or ex-smokers. As shown in **Table 2** and **Figure 1**, we identified significant evidence for linkage of COPD to chromosomes 15q14–15q25 (HLOD = 5.52), 11p15.4–11q14.1 (HLOD = 3.71), and 5q14.3–5q33.2 (HLOD = 3.49).

**Table 1:** General characteristics of the populations used in this study.

	ERF			RS	Hobbs et al., 2016	Life lines	Vlagtwedde/Vlaardingen
					
	Linkage^∗^	Exome-chip	Exome-sequence	HRC imputed	Exome-chip^∗∗^	GoNL imputed	GoNL imputed
Number	142	572	636	11,372	12,165	11,177	1,394
Age, mean(sd)	59.7 (10.9)	51.7 (14.2)	48.5 (14.0)	65.1 (9.8)	58.4 (10.3)	48.2 (11.0)	52.7 (10.2)
Female gender, %(*n*)	59.9 (85)	56.8 (325)	61.8 (393)	58.0 (6,592)	44.5 (5,410)	58.6 (6,547)	46.3 (646)
COPD cases, %(*n*)	100 (142)	10.1 (58)	9.3 (59)	14.0 (1,588)	50.6 (6,161)	14.7 (1,647)	26.9 (375)
Never smokers, %(*n*)	1.4 (2)	27.1 (155)	29.4 (187)	35.3 (4,011)	1.7 (212)	40.7 (4,549)	30.2 (421)
Ex-smokers, %(*n*)	23.2 (33)	27.8 (159)	28.8 (183)	48.8 (5,546)	49.6 (6,037)	36.7 (4,104)	33.1 (462)
Current smokers, %(*n*)	58.5 (83)	45.1 (258)	41.8 (266)	16.0 (1,815)	45.0 (5,473)	22.6 (2,524)	36.7 (511)


**^∗^Information on smoking was missing for 16.9% (24) participants; ^∗∗^Full dataset reported in the Hobbs et al. (2016) meta-analysis. Information on smoking was missing for 3.6% (443) participants. COPD, chronic obstructive pulmonary disease; ERF, Erasmus Rucphen Family; RS, Rotterdam study; GoNL, genome of the Netherlands*.*

**Table 2:** Genome wide significant (HLOD>3.3) results of linkage analysis in the ERF study.

Cytogenetic location^∗^	Start SNP	End SNP	SNP with highest HLOD	Start position^#^	End position^#^	Dominant model HLOD	Recessive model HLOD
15q14–15q25	rs2004175	rs1402760	rs383902	39039593	79146817	4.24	5.52
11p15.4–11q14.1	rs1609812	rs7102569	rs626333	5247141	79184899	2.61	3.71
5q14.3–5q33.2	rs1366133	rs1432812	rs1154308	91114584	155274700	2.65	3.49


**^∗^Region under the linkage peak; Start SNP – single nucleotide polymorphism (SNP) at the beginning of the corresponding region; End SNP – SNP at the end of the corresponding region; HLOD, heterogeneity log of odds score; ^#^Corresponding to the region from base to base of the linkage peak, based on the hg19 assembly*.*

**FIGURE 2:**
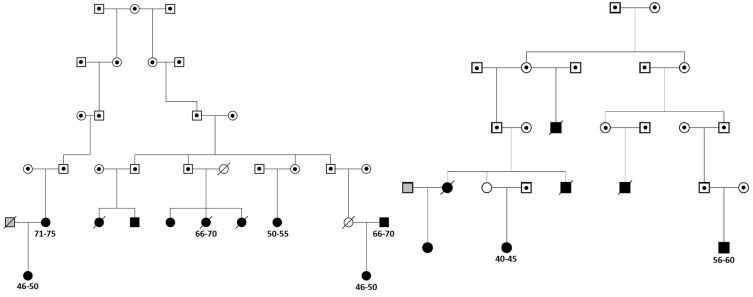
The two sub-families contributing most to the linkage peak on chromosome 11. Squares represent males and circles females. Cases are denoted in black, known controls are denoted in gray and the family members for which we do not have chronic obstructive pulmonary disease (COPD) information are denoted in white. Family members with dot in the middle are not included in Erasmus Rucphen family (ERF) study and for them only pedigree information was available. Deceased family members are crossed. For cases with exome-sequence data used in the sharing analysis information on 5-year age range (in years) is provided.

We next searched for rare, deleterious and shared variants by most (>50%) of the affected family members in the three identified regions mentioned above. In the linked regions of chromosomes 5 and 15 we could not identify any variants that passed mentioned filtering criteria. For the linked region on chromosome 11, we identified two families that were contributing most (LOD > 1) to the linkage score (**Figure 2**). Exome-sequence data were available for 8 of 17 COPD cases from these two families. We identified four missense variants including rs116243978 (*AHNAK*), rs35169799 (*PLCB3*), rs141159367 (*SLC22A11*), and rs146043252 (*MTL5*), shared among five of the eight affected family members (**Table 3**). Each of these variants was predicted to be highly pathogenic (CADD > 15, PolyPhen > 0.98) which suggests their relevance for the disease development. Of these four variants, one (rs141159367 in *SLC22A11)* showed a significant association (OR = 1.87, *P* = 0.002) with COPD in the meta-analysis (**Table 4**). The variant rs146043252 in *MTL5* showed a nominal association signal (OR = 1.66, *P* = 0.04).

**Table 3:** Deleterious variants from chromosome 11q (missense, stop codon or CADD > 15) with a frequency in the 1000 genomes <0.05 that are shared by at least 5 cases.

Gene	Variant	1 KG MAF	ERF MAF	Cytogenetic band	Position (hg19)	A1	A2	Carrier-HET	Carrier-HOM	Function	CADD	PolyPhen
*AHNAK*	rs116243978	0.005	0.04	11q12.3	62286165	G	C	5/8	0/8	Missense	15.55	1
*PLCB3*	rs35169799	0.023	0.08	11q13.1	64031241	T	C	6/8	1/8	Missense	15.73	0.982
*SLC22A11*	rs141159367	0.0006	0.04	11q13.1	64323476	T	C	5/8	1/8	Missense	18.25	1
*MTL5*	rs146043252	0.0002	0.04	11q13.3	68478487	G	A	5/8	0/8	Missense	21	1


**1 KG MAF – minor allele (A1) frequency (MAF) in 1000 Genomes – EUR; A1: effect allele; A2: alternative allele; Carrier-HET: number of heterozygote carriers within the 8 COPD cases of the two top contributing families of the chromosome 11 region; Carrier-HOM: number of homozygote carriers within the 8 COPD cases; Function: predicted function of the variant; CADD: Combined Annotation Dependant Depletion score (>15 considered deleterious); PolyPhen: probability that variant is damaging*.*

**Table 4:** Results of association analysis with COPD.

Gene	Variant	β	OR	SE	*P*	*N*
*AHNAK*	rs116243978	0.14	1.15	0.18	0.422	13,402
*PLCB3*	rs35169799	0.05	1.05	0.04	0.247	36,948
*SLC22A11*	rs141159367	0.63	1.87	0.20	**0.002**	18,562
*MTL5*	rs146043252	0.51	1.66	0.25	**0.044**	12,050


**Meta-analysis of the ERF exome-sequence, ERF exome-chip, Rotterdam Study – HRC, Hobbs et al. (2016) exome-chip, LifeLines – GoNL, and Vlagtwedde/Vlaardingen cohort – GoNL results. In bold: significant results. β: Regression coefficient estimates from logistic regression model regressing COPD status on indicated variants, corrected for sex, age, and smoking; OR, odds ratio; SE, standard error of the effect; P*, p-value of the significance; N-sample size used in the analysis.*

## Discussion

In this study, we found significant evidence for extensive linkage of COPD to the chromosomes 15q14–15q25 (40.1 Mb), 11p15.4–11q14.1 (73.9 Mb), and 5q14.3–5q33.2 (64.1 Mb). We were able to identify four rare and predicted pathogenic variants under the chromosome 11 peak, in plausible genes (*AHNAK, PLCB3, SLC22A11*, and *MTL5*), shared by at least five family members. One of these four variants, i.e., rs141159367 in *SLC22A11*, was significantly associated with COPD in 9,888 cases and 27,428 controls (*P* = 0.002) while another variant (rs146043252 in *MTL5*) showed nominal association with COPD (*P* = 0.04).

The finding of our family-based linkage analysis aligns with that of large scale GWASs implicating the *CHRNA3/5-CHRNB4*, and *IREB2* region on chromosome 15q25 in COPD development. This region is also associated with lung cancer, peripheral arterial disease, nicotine addiction and smoking quantity (Thorgeirsson et al., 2008). The evidence in the literature on the role of smoking in the genetic risk of COPD thus far is controversial. On one hand, there is evidence to support that the variants in this region, although implicated in both lung disease and smoking behavior, are associated with COPD susceptibility, independently of cigarette smoke exposure (Hardin and Silverman, 2014). On the other hand, in a previous study we show that two variants, previously associated with COPD in the *CHRNA3/5* locus, were associated with lung function measurements in ever-smokers, but not in never-smokers (van der Plaat et al., 2017), which is in line with the only longitudinal study on the relation between the nicotine receptor variant and annual lung function decline (Budulac et al., 2012). That study shows that carriers of the nicotinic receptors variants are significantly less able to quit smoking, leading to the lung function decline and, subsequently to COPD. Similarly, for the chromosome 5 linked region, we could not observe any shared rare variant. This region, known for its associations with pulmonary function and airflow obstruction (Hancock et al., 2010; Wilk et al., 2012) was recently associated with COPD by the largest GWAS to date (Hobbs et al., 2017). The *HTR4* gene in 5q32 encodes a serotonin receptor involved in depression and is strongly expressed in respiratory complex neurons (Manzke et al., 2003).

However, the functional variants in these regions have still not been confirmed. In our families, we could not identify rare damaging variants shared between the cases in this region. This may be explained if rare intronic regulatory variants play a key role, which we could not investigate using the exome data. It is unlikely that these linkage peaks are attributed to the common variants which have small effects identified in GWASs, given the very strong evidence for linkage of this region to COPD. Future studies using whole-genome sequencing should investigate this region further, ideally in never smokers. This emphasizes the need for integration of available genomic information into more focused, candidate-gene based efforts to disentangle the functional role of the chromosome 5 and 15 regions.

In the identified region of chromosome 11 we were able to pinpoint four strong candidate genes for the association with COPD, i.e., *SLC22A11*, *AHNAK, PLCB3*, and *MTL5*. The most interesting finding is the rare variant in *SLC22A11* (solute carrier family 22 member 11), which encodes an integral membrane protein and part of the family of OATs, known to mediate the absorption and elimination of endogenous and exogenous organic anions and as such, are involved in the pharmacokinetic, pharmacodynamic and safety profiles in a wide range of drugs (Bosquillon, 2010). *SLC22A11* (OAT4) is mainly expressed in kidney and placenta. However, it is also shown to be expressed in lung tissue, fibroblasts and T-lymphocytes (*P <* 5 × 10^-7^), among other tissues/cells reported in the Gene network (Fehrmann et al., 2015). In addition, *in vitro SLC22A11* mRNA was absent in normal human bronchial epithelial cells, but highly expressed in other bronchial cells models comprising transformed cells (Endter et al., 2009). *SLC22A11* in particular is known to be a drug target for probenecid, a *SLC22A11* inhibitor, used in the gout prevention and to increase antibiotic blood levels, yet its direct role in lung disease treatment is still unknown (Bosquillon, 2010).

Our linkage analysis yielded different regions compared with those identified earlier. However, the fact that both *SLC22A11* and *MTL5* variants were associated with COPD in our meta-analysis confirms their role in COPD and makes them even more interesting candidates. *MTL5* (metallothionein-like protein 5) encodes testis expressed metallothionein like proteins (TESMIN*).* They are highly conserved, low-molecular-weight cysteine-rich proteins induced by and binding to heavy metal ions, and they do not have enzymatic activity. They play a central role in the regulation of cell growth and differentiation, and are involved in spermatogenesis, differentially regulating meiosis in male and female cells (Olesen et al., 2004). *MTL5* was shown to be involved in nicotinate and nicotinamide metabolism and is also expressed in fibroblasts and lung tissue (*P <* 7 × 10^-29^), based on the Gene network (Fehrmann et al., 2015). Metallothioneins were additionally shown to protect cells against oxidative stress damage and participate in differentiation, proliferation and/or apoptosis of normal and lung cancer cells (Werynska et al., 2015).

The main strength of our study is the genetically isolated family-based population, which can display increased frequencies of some variants found at very low proportions in panmictic populations. This allowed us to perform a genome-wide linkage scan and identify rare coding variants. However, even though we identified linkage of three regions to COPD, a limitation of our study is the low power to explain the peaks at chromosomes 5 and 15, possibly due to the use of exome data. As intronic regulatory variants may play a significant role, in the future, faster and cheaper whole-genome sequencing will allow us to improve identification of rare variants and our understanding of their involvement in COPD. As our sample consists of high percentage of current or ex-smokers, it is possible that we are demonstrating genetic effects on smoking which further affects the development of COPD. Nevertheless, we were able to demonstrate a positive association, independent of smoking, of two variants in the association meta-analysis comprising 9,888 cases and 27,060 controls. Yet, studies with very large sample sizes utilizing mediation or mendelian randomization techniques are needed to disentangle these relationships and confirm our results in the general population.

## Conclusion

Using the powerful genome-wide linkage scan in a Dutch genetic isolate, we have confirmed the implication of the 15q25 region in COPD and identified regions at chromosomes 5 and 11. Within the region on chromosome 11 we identified four deleterious rare variants shared between most of the affected family members in *AHNAK, PLCB3, SLC22A11* and *MTL5.* The variants in *SLC22A11* and *MTL5* were significantly associated with COPD in our meta-analysis. Further studies pooling large sample sizes could confirm the role of the identified rare variants at chromosome 11 in the general population. Similarly, large studies utilizing whole-genome sequencing should further investigate the role of linked regions in chromosomes 5 and 15 in COPD.

## Author Contributions

IN, NA, NT, LL, JV, DvdP, BH, DQ, and MC were involved in the analysis of the data. IN, JV, DvdP, CCvD, DvdP, HB, CMvD, and NA contributed to the conception and design of this work and were involved in the interpretation of the results. IN, JV, DvdP, LL, GB, and DvdP were involved in data collection/preparation. All authors were involved in writing and critically revising the manuscript, approved the final manuscript, and agreed to be accountable for it.

## Conflict of Interest Statement

The authors declare that the research was conducted in the absence of any commercial or financial relationships that could be construed as a potential conflict of interest.

## Footnotes

1 https://www.ebi.ac.uk/ega/home

2 http://snp.gs.washington.edu/SeattleSeqAnnotation138/

## Abbreviations

AAT: Alpha-1-antitrypsin
ATS: American thoracic society
CADD: Combined Annotation Dependent Depletion
COPD: chronic obstructive pulmonary disease
CT: computed tomography scan
ERF: Erasmus Rucphen Family
ERS: European Respiratory Society
FEV_1_: forced expiratory volume in one second
FVC: forced vital capacity
GoNL: genome of the Netherlands
GWAS: genome-wide association study
HLOD: heterogeneity log of odds score
HRC: haplotype reference consortium
KEGG: Kyoto Encyclopedia of Genes and Genomes
LLS: lifelines study
LOD: logarithm of the odds
MAF: minor allele frequency
OATs: organic anion transporters
QC: quality control
RS: Rotterdam Study
SNVs: single nucleotide variants
VlaVla: Vlagtwedde/Vlaardingen-study
