# Supplementary material for: A Genome-Wide Linkage Study for Chronic Obstructive Pulmonary Disease in a Dutch Genetic Isolate Identifies Novel Rare Candidate Variants
Source: Front Genet. 2018 Apr 19;9:133. doi: 10.3389/fgene.2018.00133 (PMC5916965; doi:10.3389/fgene.2018.00133)
Supplement: Supplementary file 1 [file Image_1.pdf]

Supplementary Material

A genome-wide linkage study for chronic obstructive pulmonary disease in a Dutch genetic isolate identifies novel rare candidate variants

Ivana Nedeljkovic, Natalie Terzikhan, Judith M. Vonk, Diana A. van der Plaat, Lies Lahousse, Cleo C. van Diemen, Brian D. Hobbs, Dandi Qiao, Michael H. Cho, Guy G. Brusselle, Dirkje S. Postma, H. Marike Boezen, Cornelia M. van Duijn, Najaf Amin**

*** Correspondence:** Najaf Amin: n.amin@erasmusmc.nl

Supplementary Figures

Figure S1. All 27 sub-families used in the linkage analysis. Squares represent males and circles females. Cases are denoted in black, known controls are denoted in grey and the family members for which we do not have COPD information are denoted in white. Family members with dot in the middle are not included in ERF study and for them only pedigree information was available. Deceased family members are crossed. Family 3 and 4 are the ones contributing the most to the linkage peak at chromosome 11 (Figure 2).
