# Supplementary material for: A Genome-Wide Linkage Study for Chronic Obstructive Pulmonary Disease in a Dutch Genetic Isolate Identifies Novel Rare Candidate Variants
Source: Front Genet. 2018 Apr 19;9:133. doi: 10.3389/fgene.2018.00133 (PMC5916965; doi:10.3389/fgene.2018.00133)

Family 1

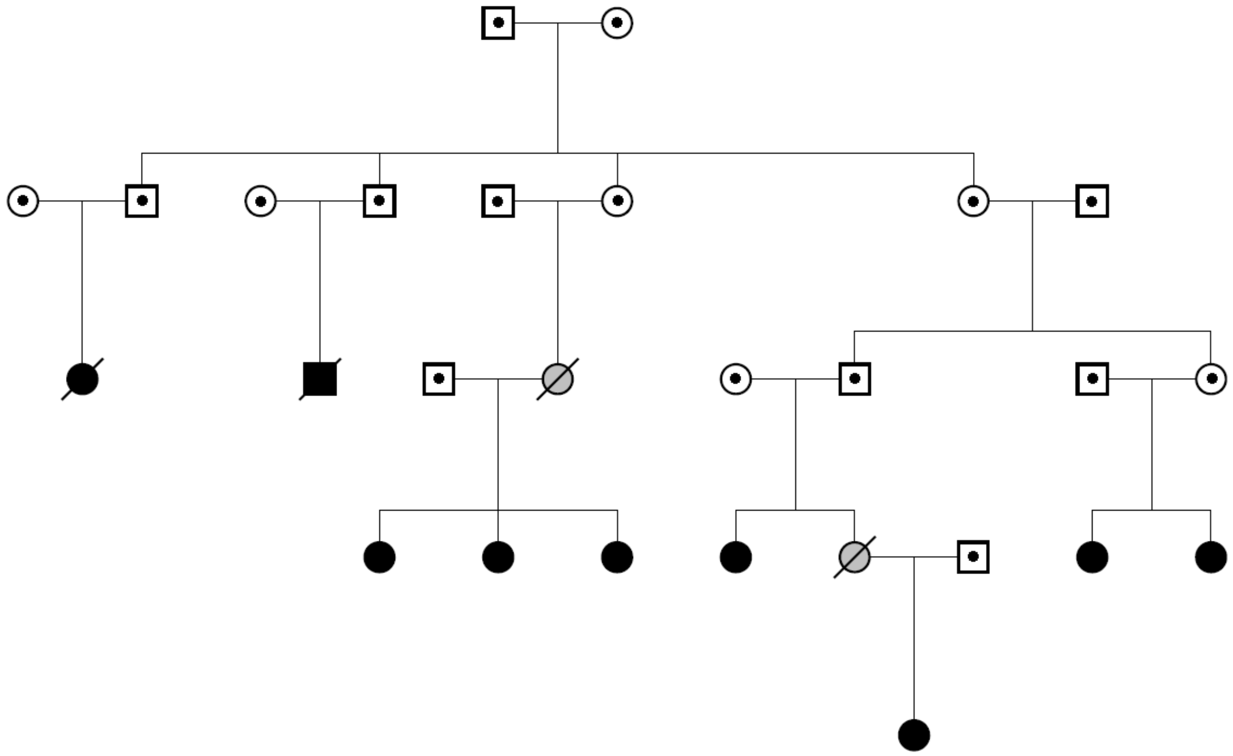

Family 2

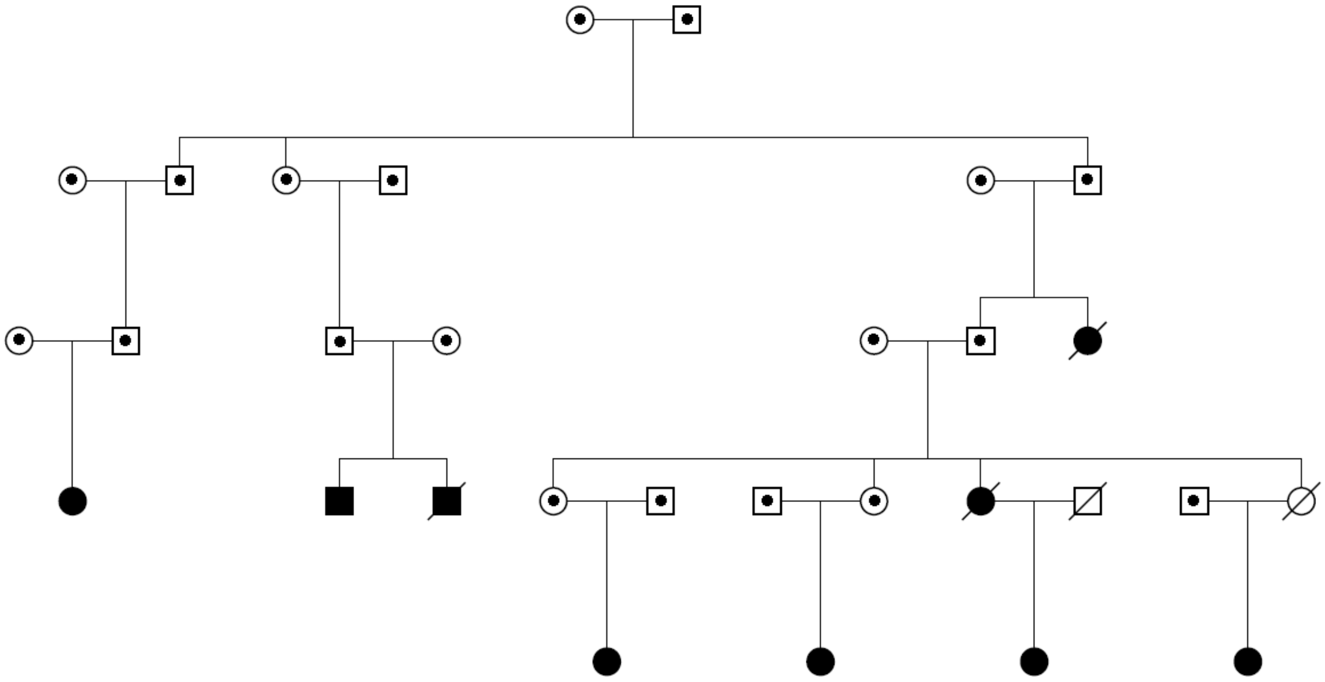

Family 3

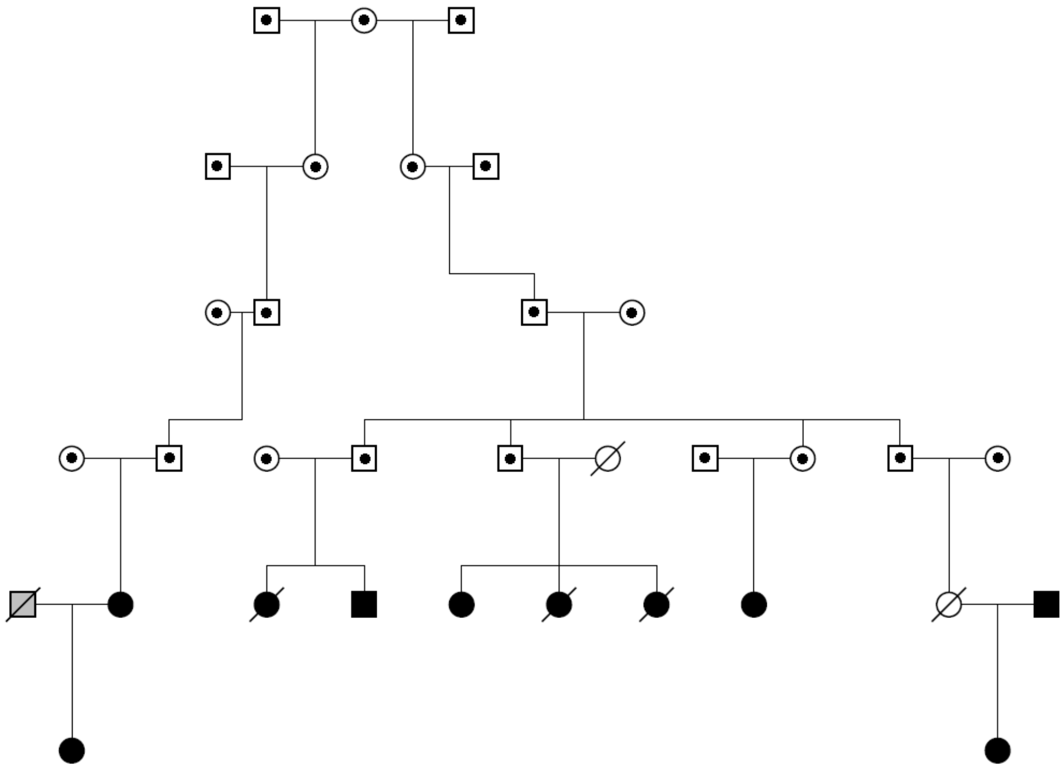

Family 4

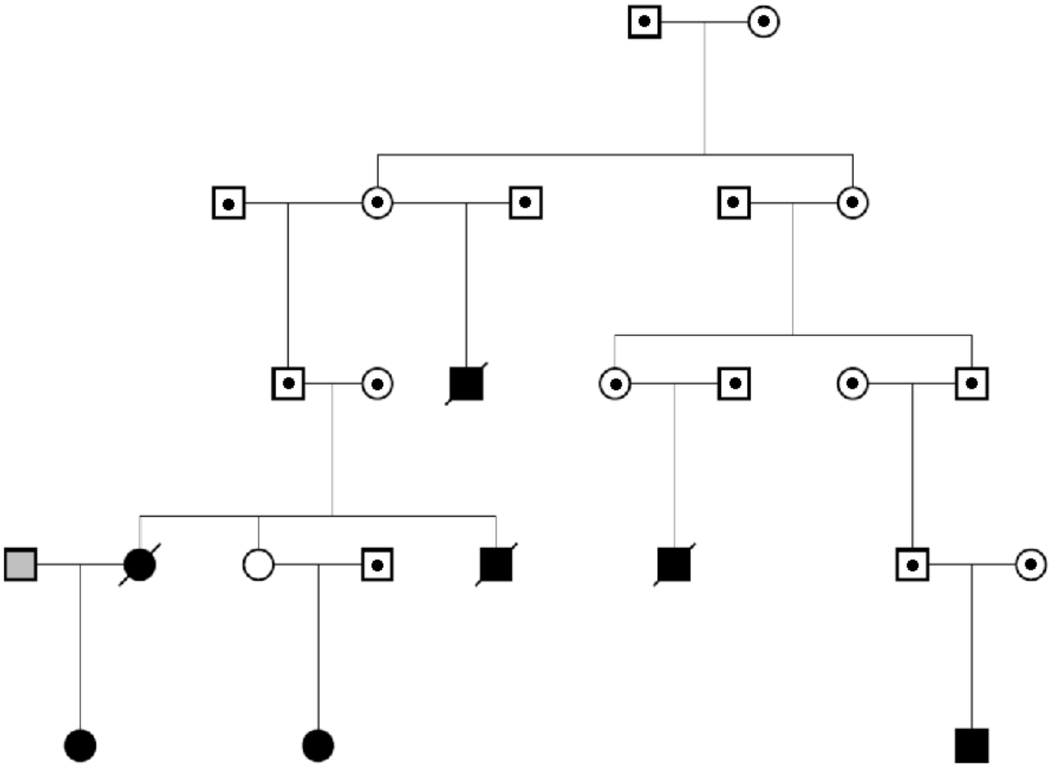

Family 5

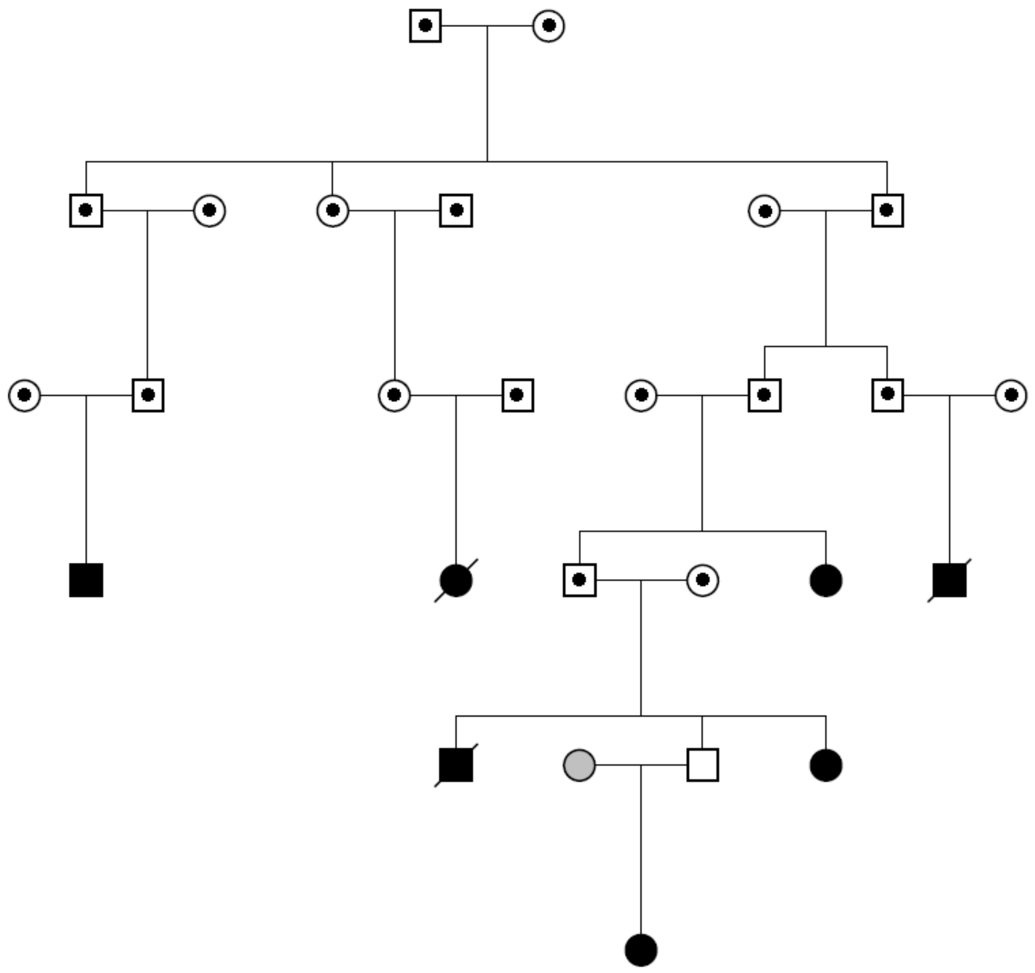

Family 6

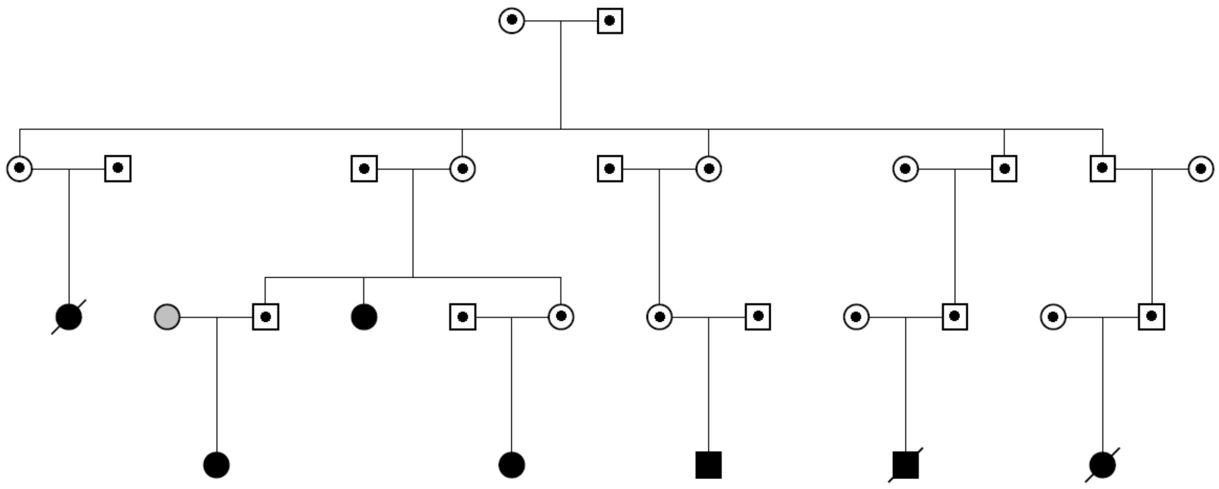

Family 7

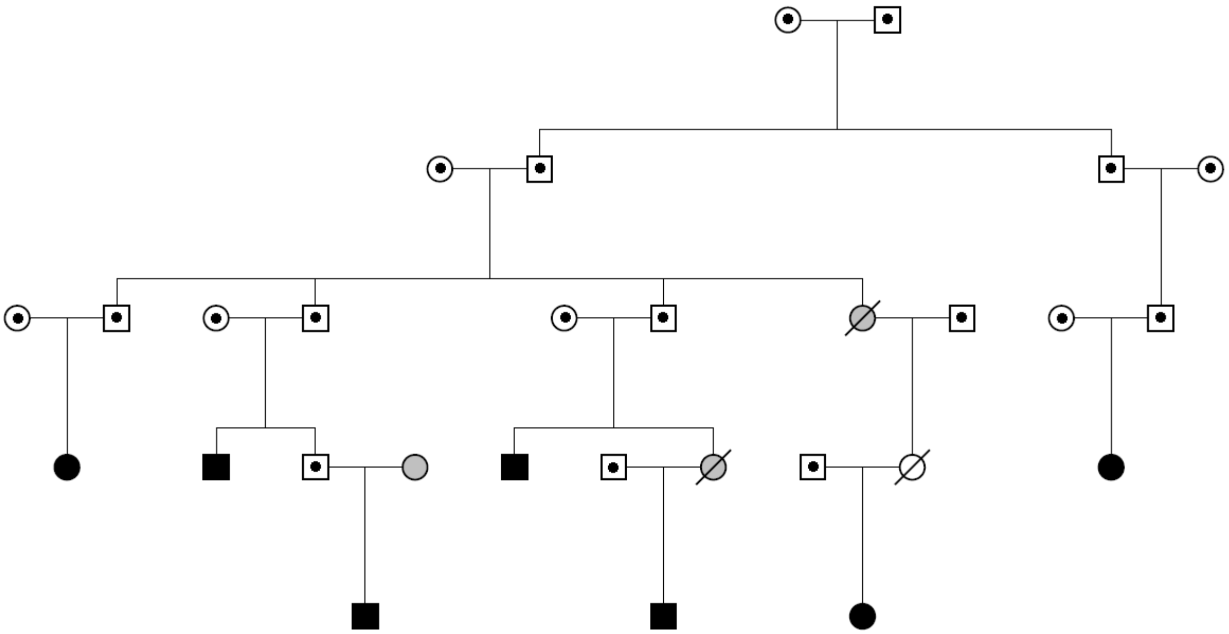

Family 8

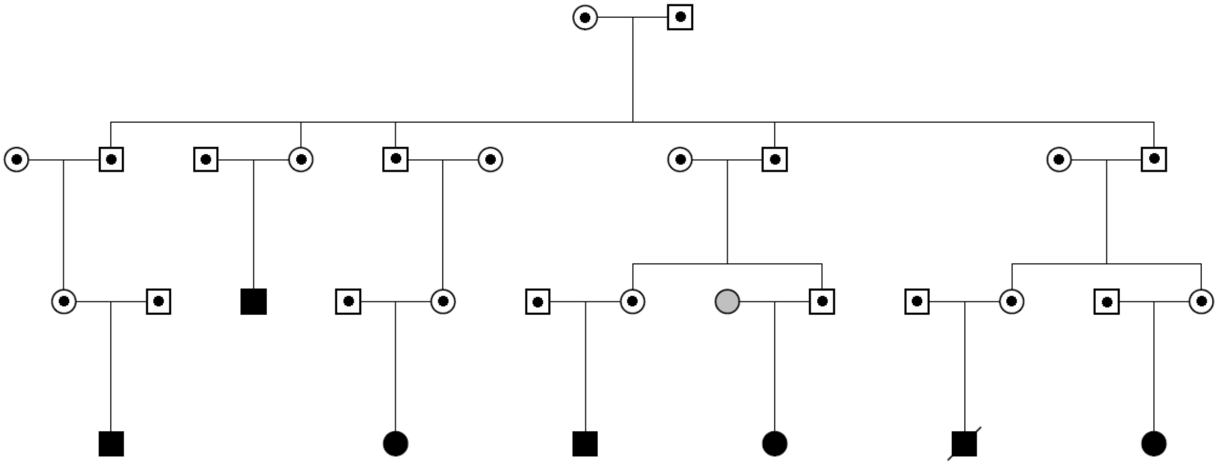

Family 9

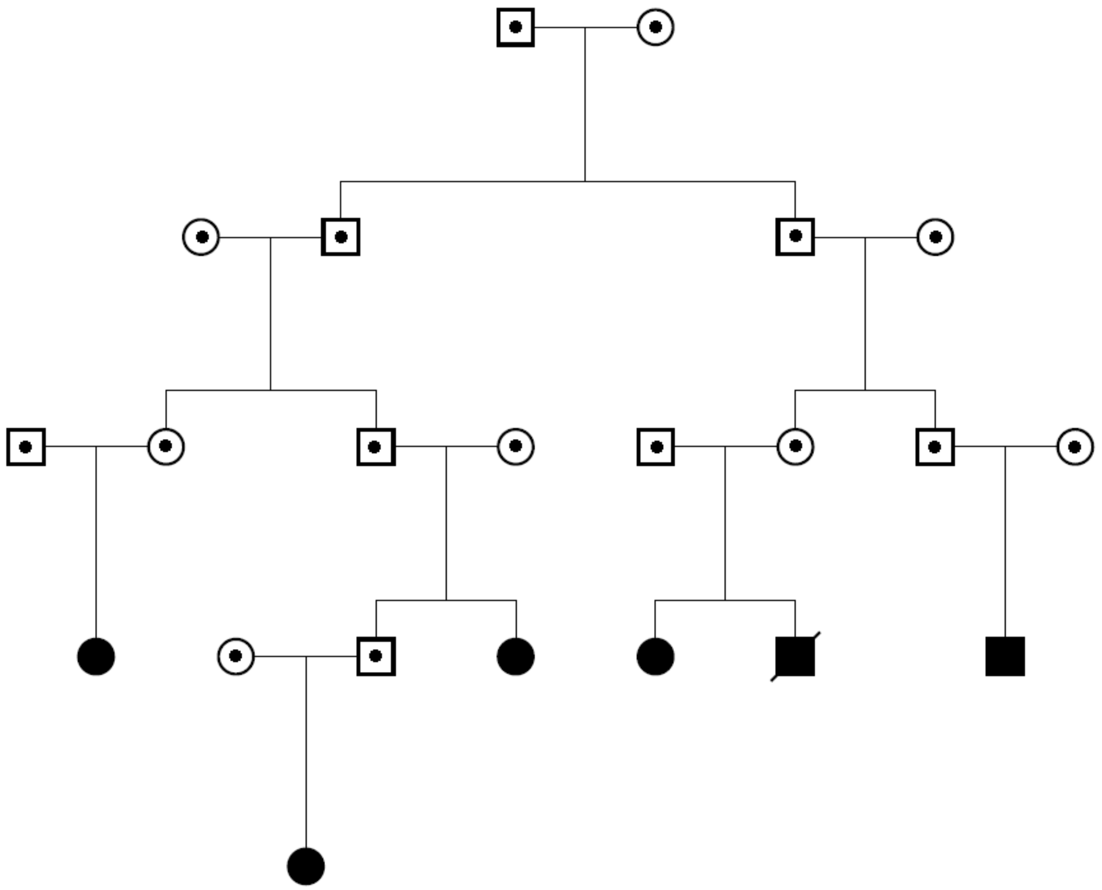

Family 10

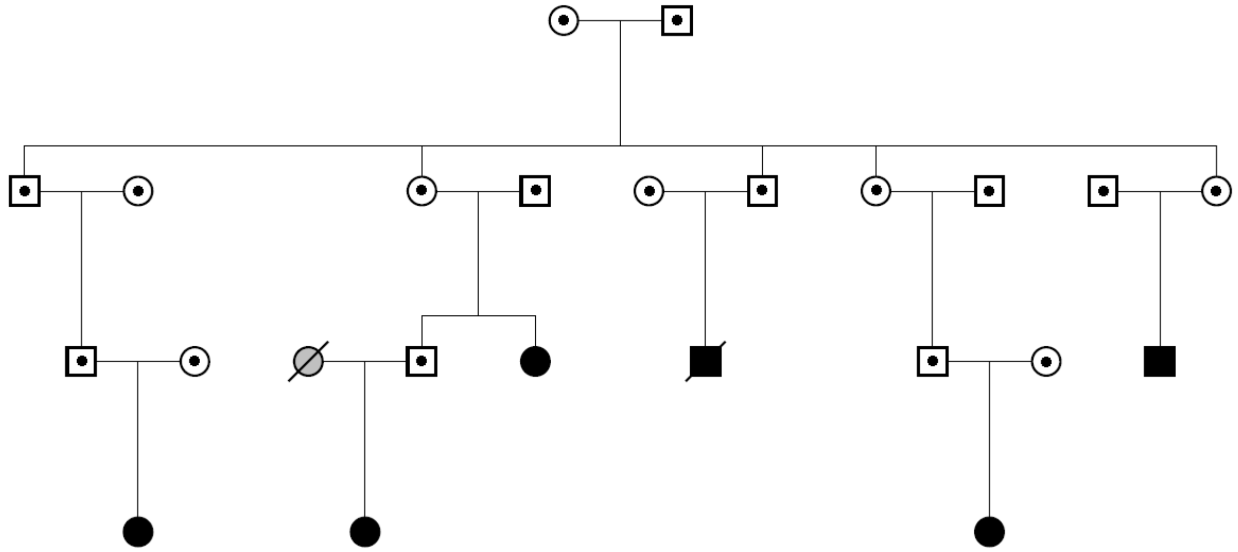

Family 11

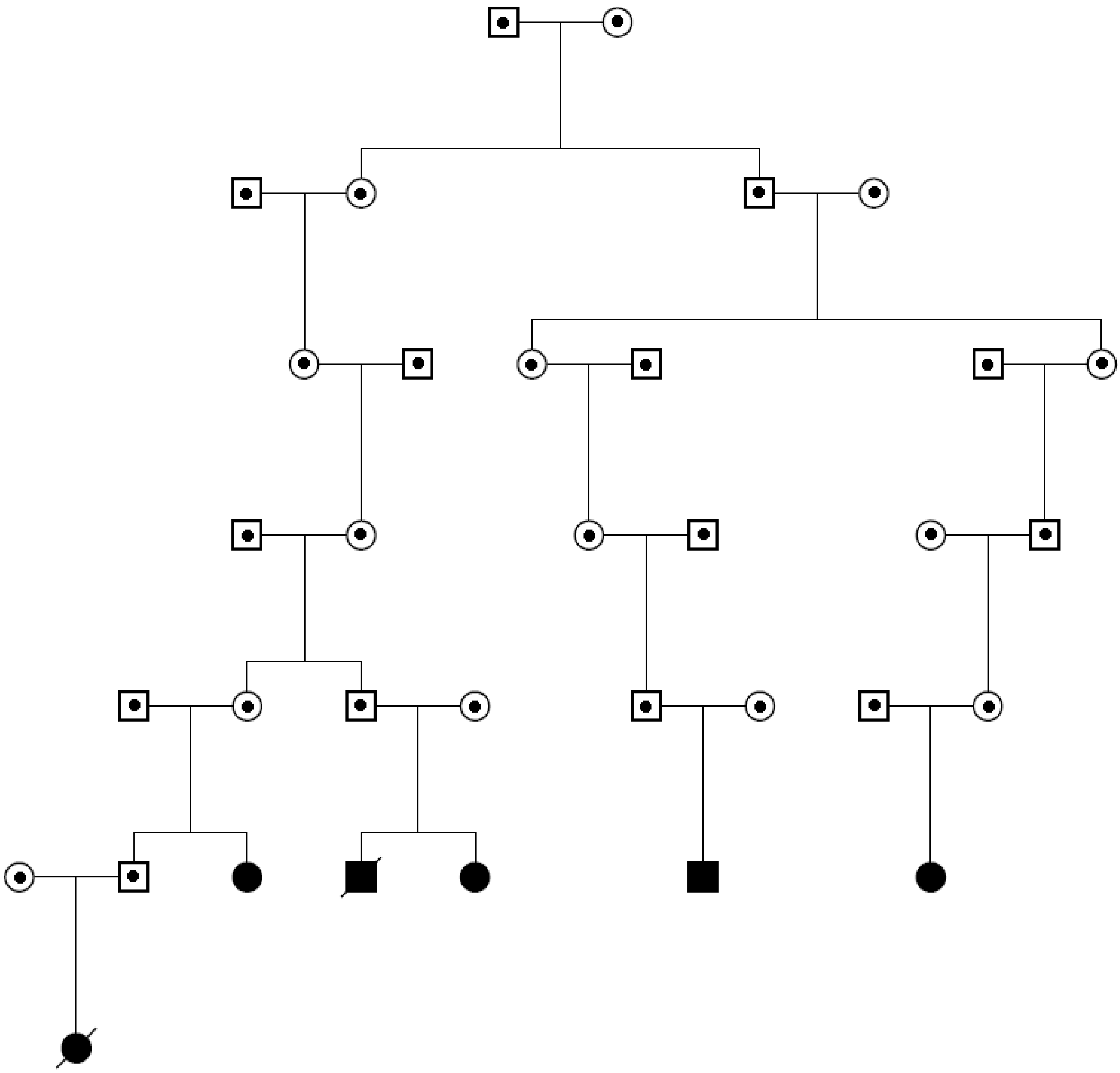

Family 12

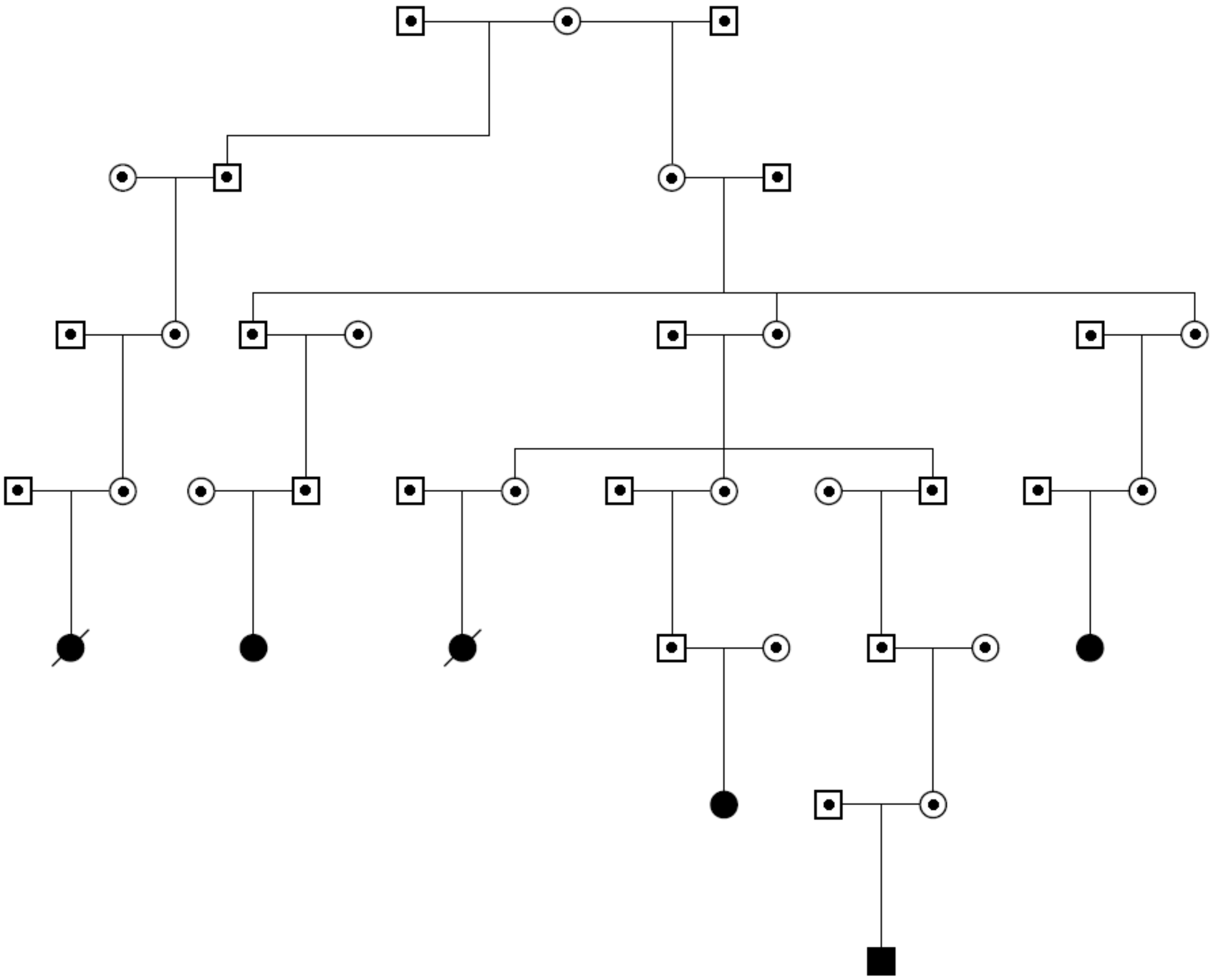

Family 13

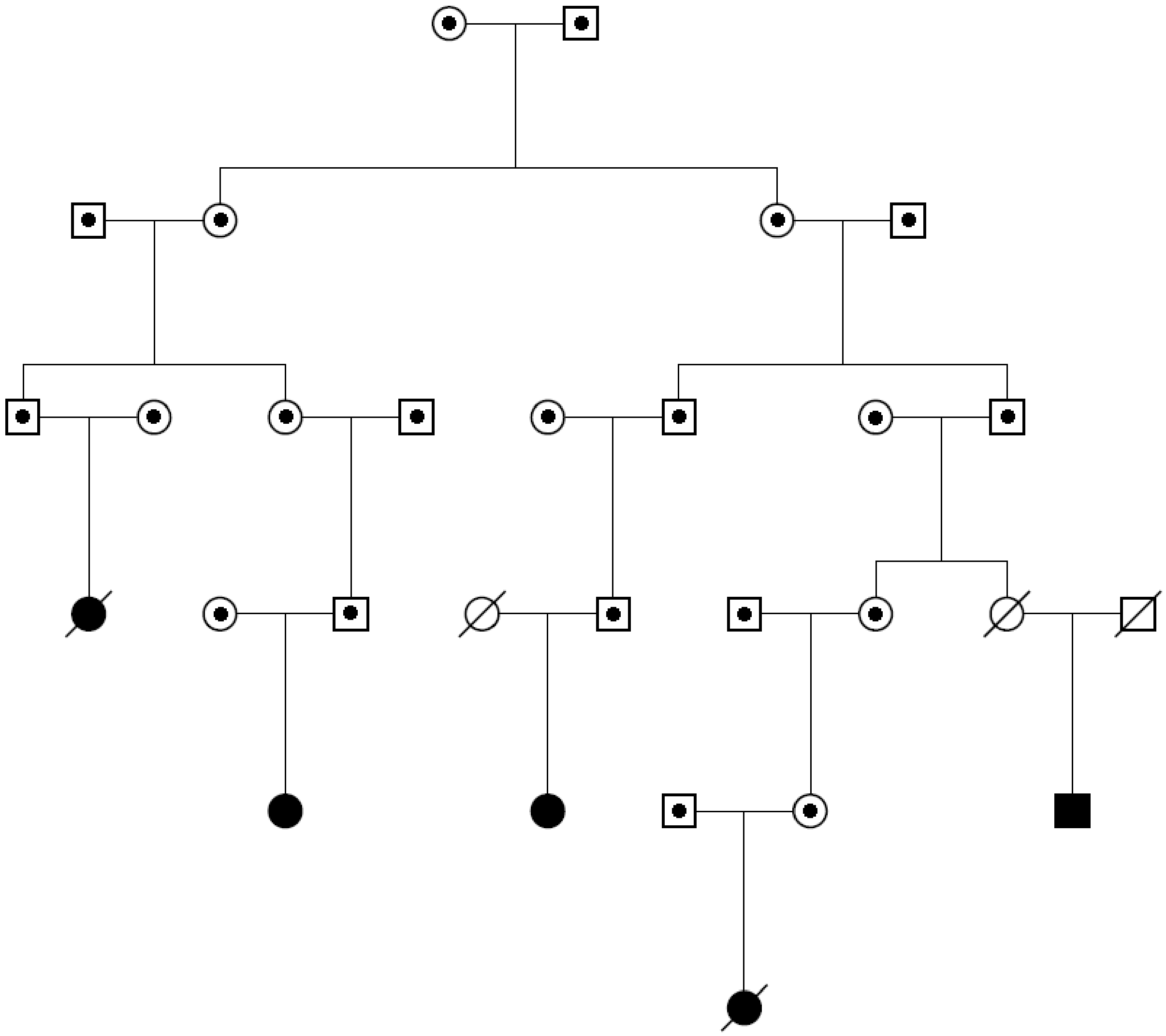

Family 14

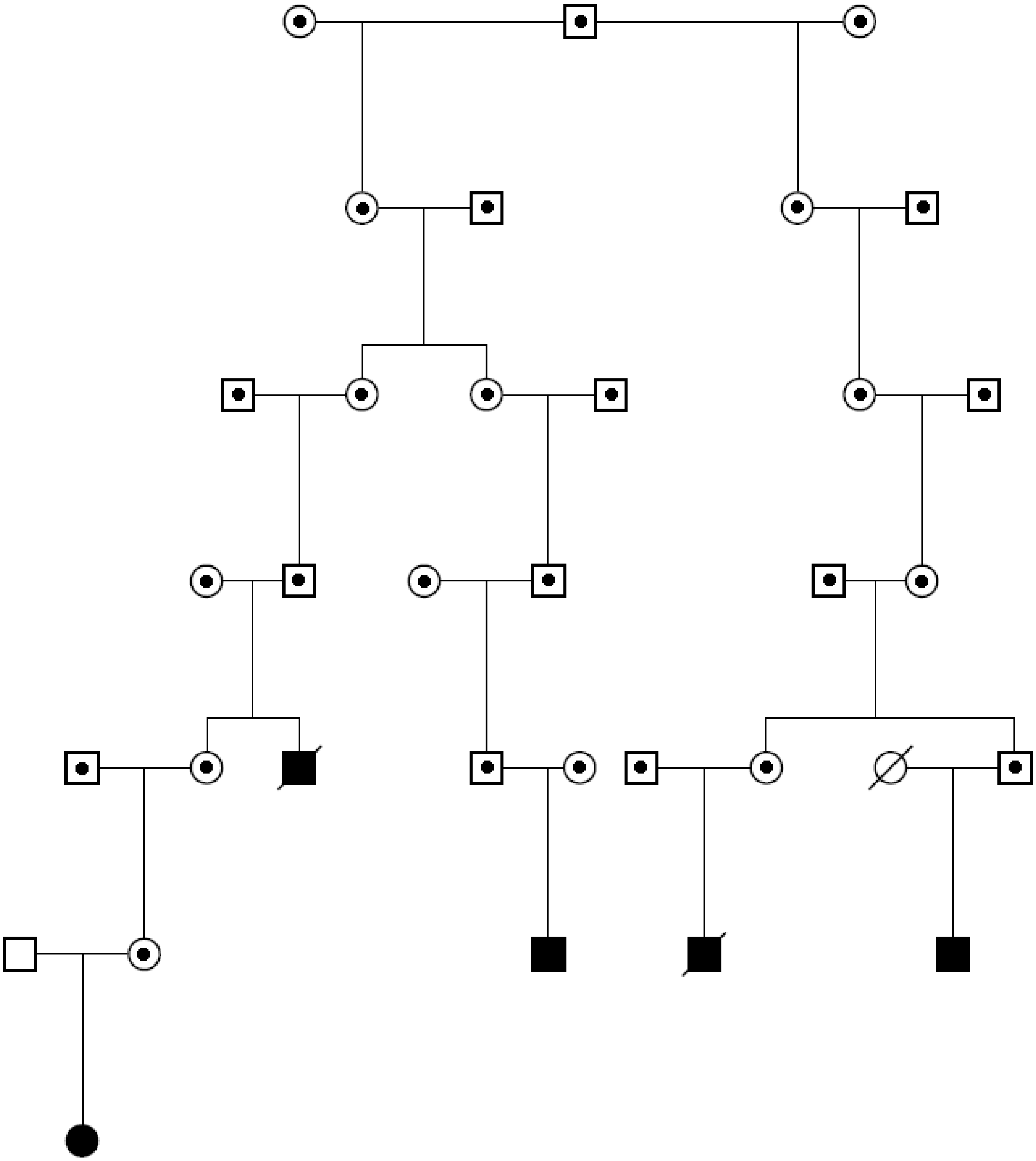

Family 15

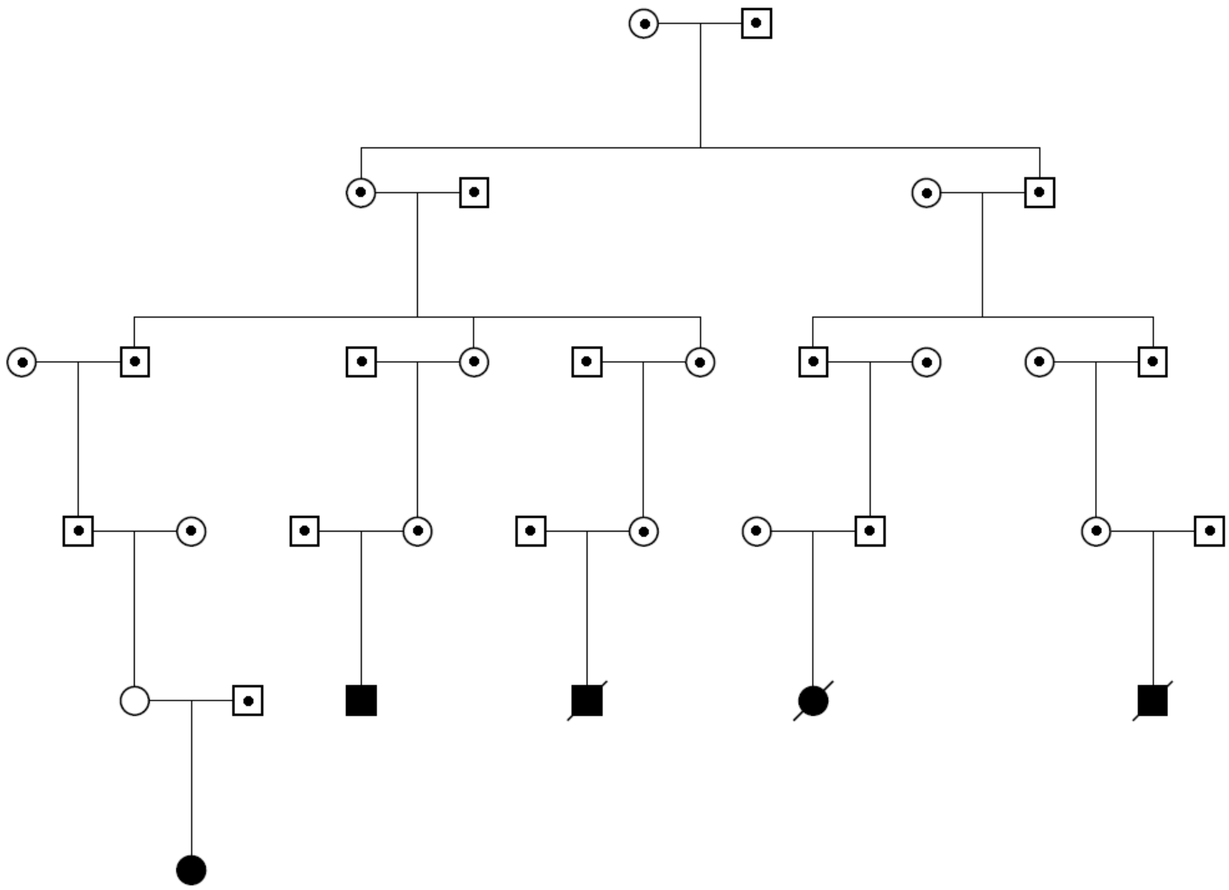

Family 16

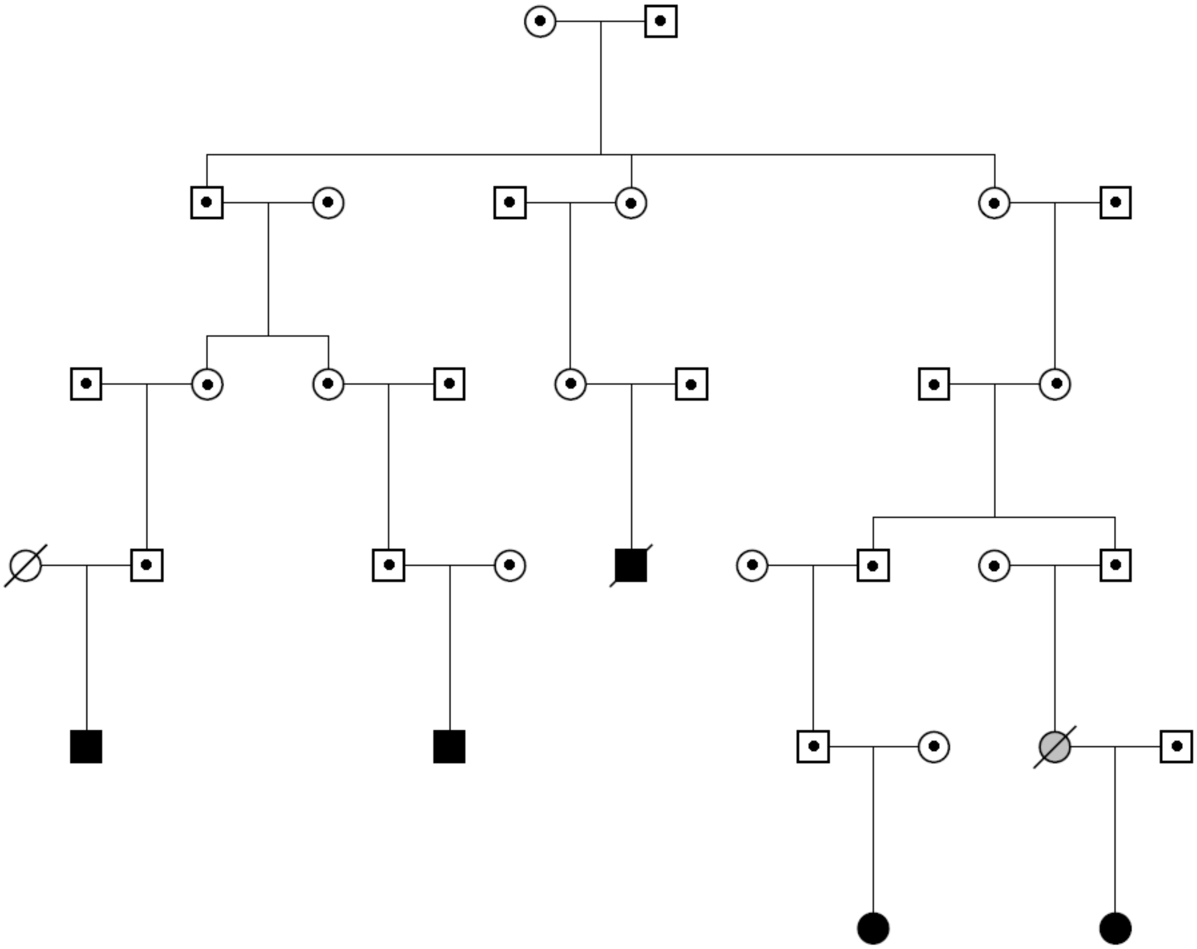

Family 17

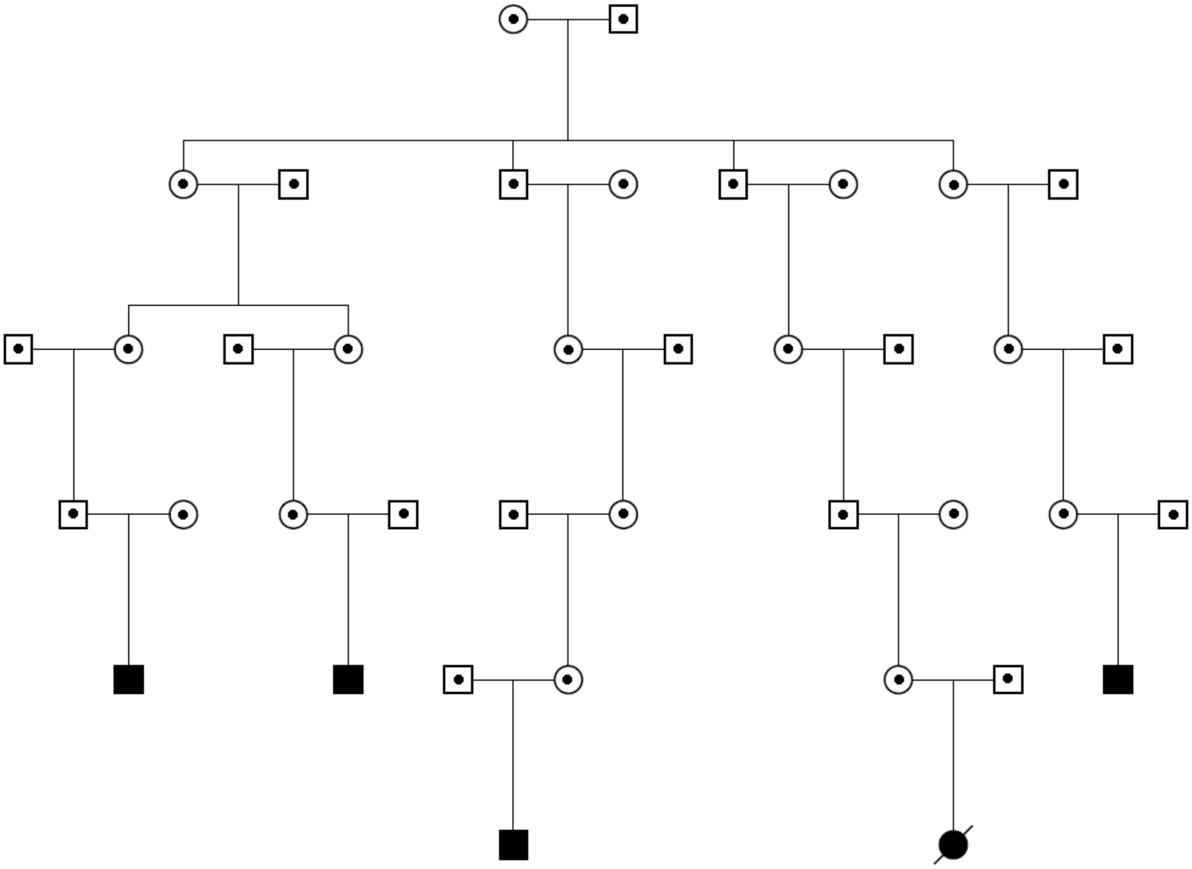

Family 18

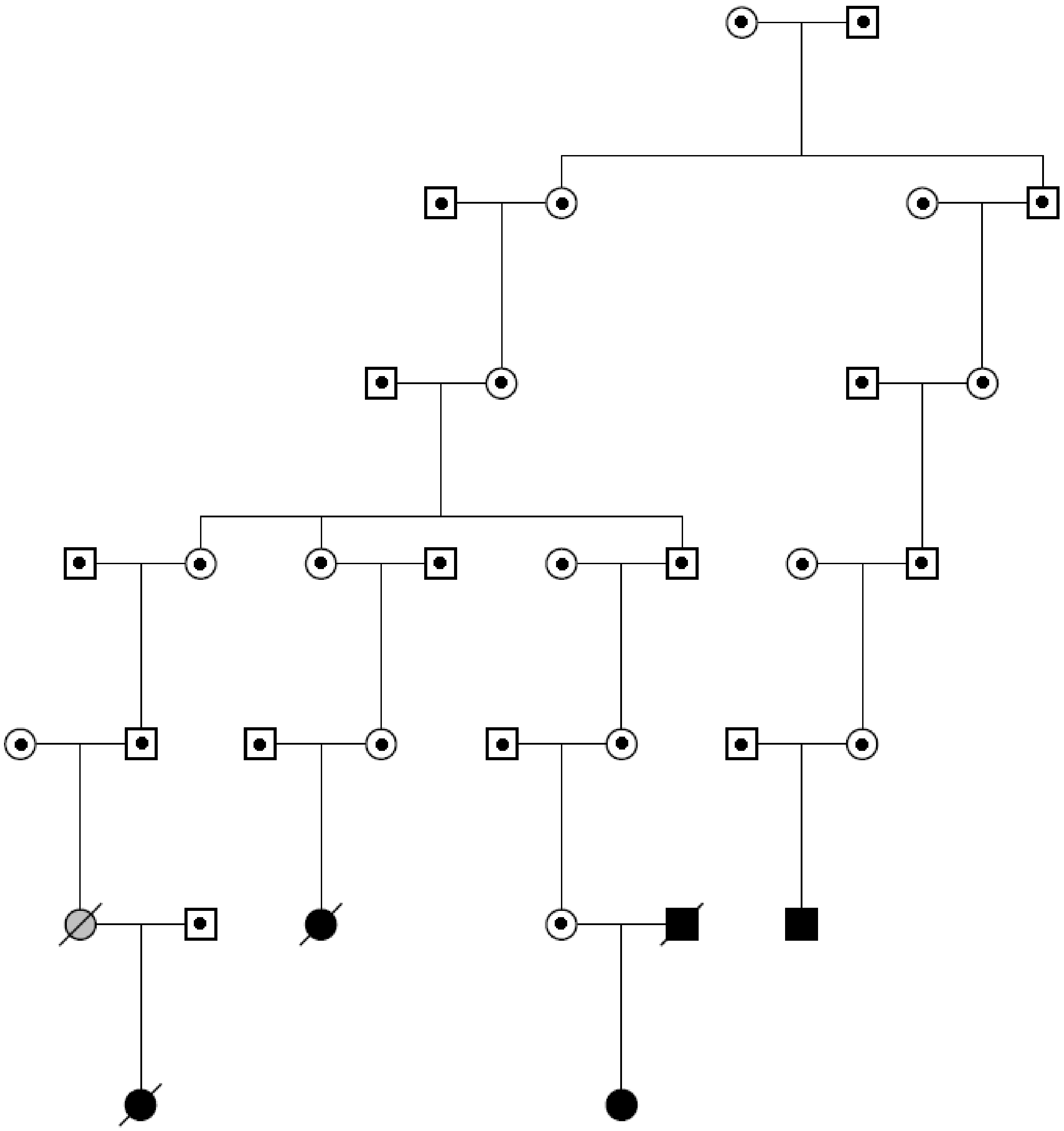

Family 19

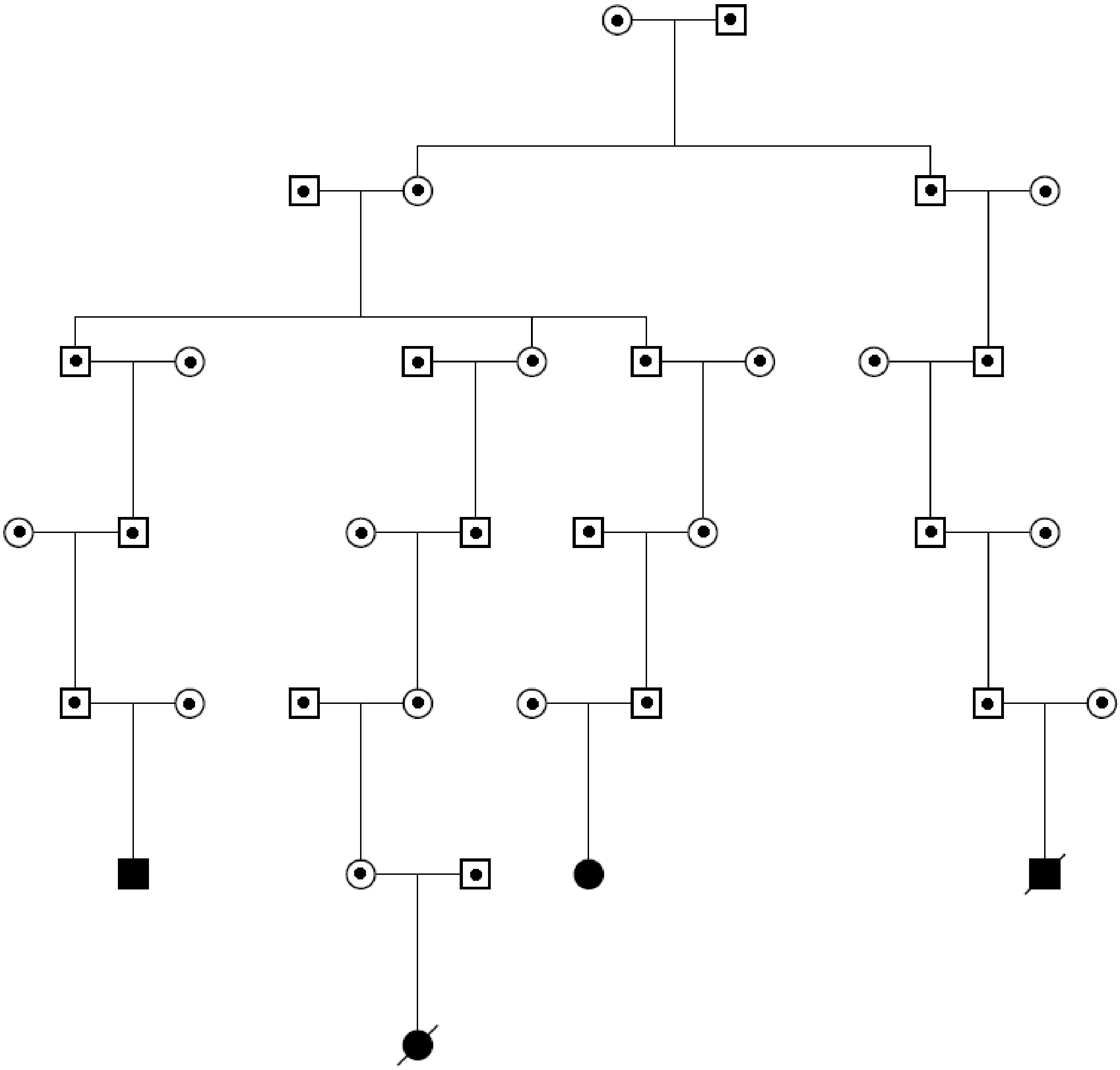

Family 20

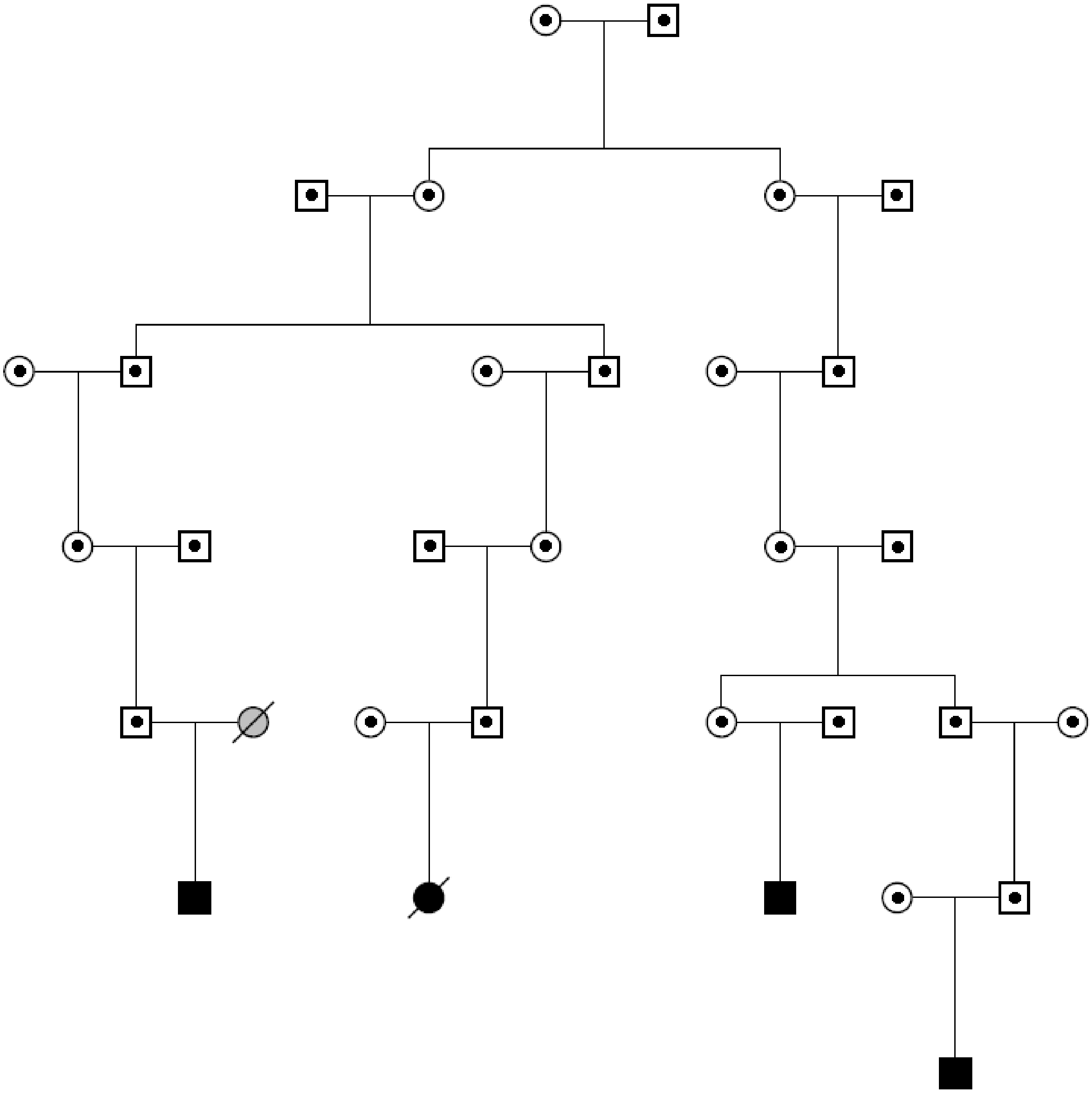

Family 21

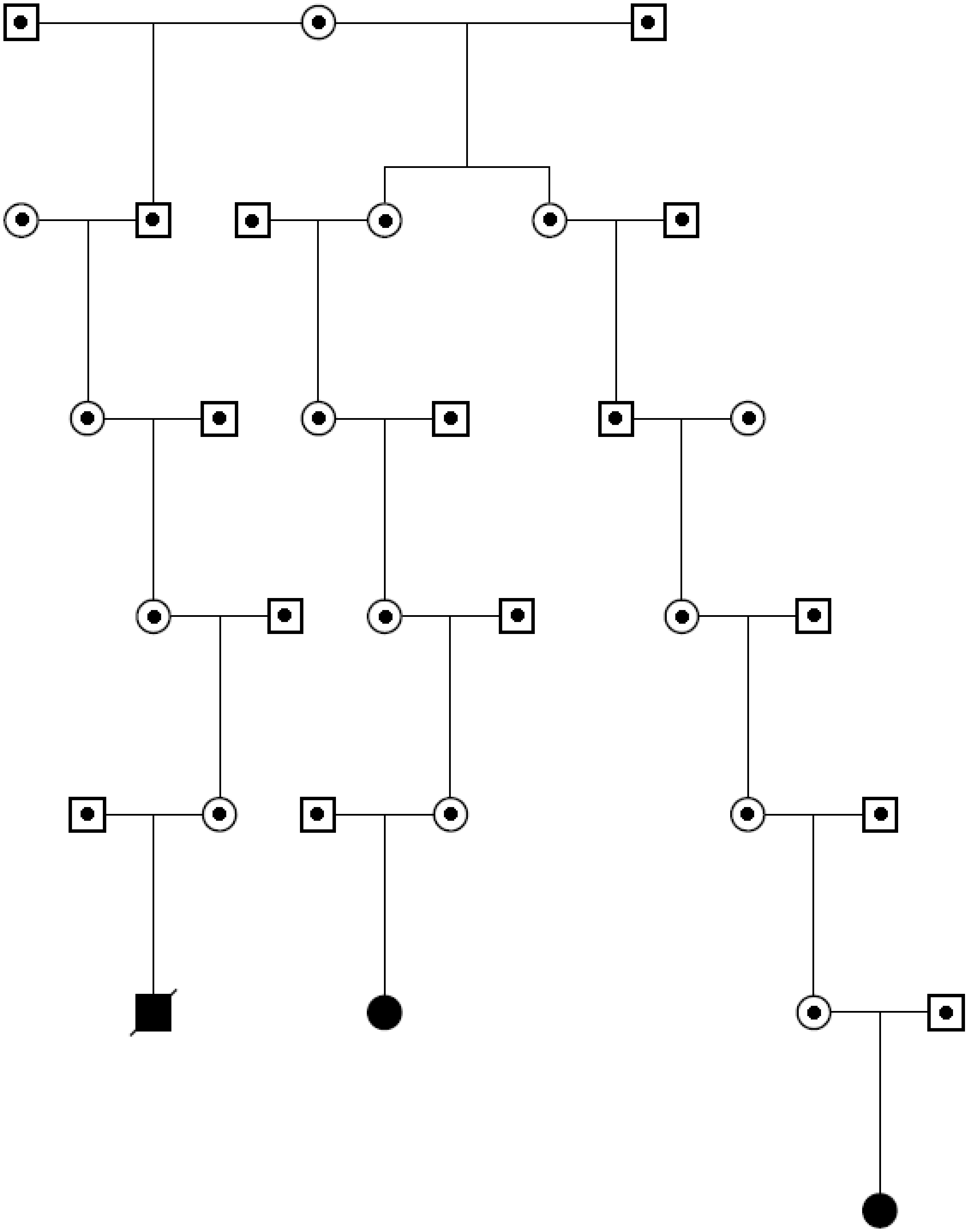

Family 22

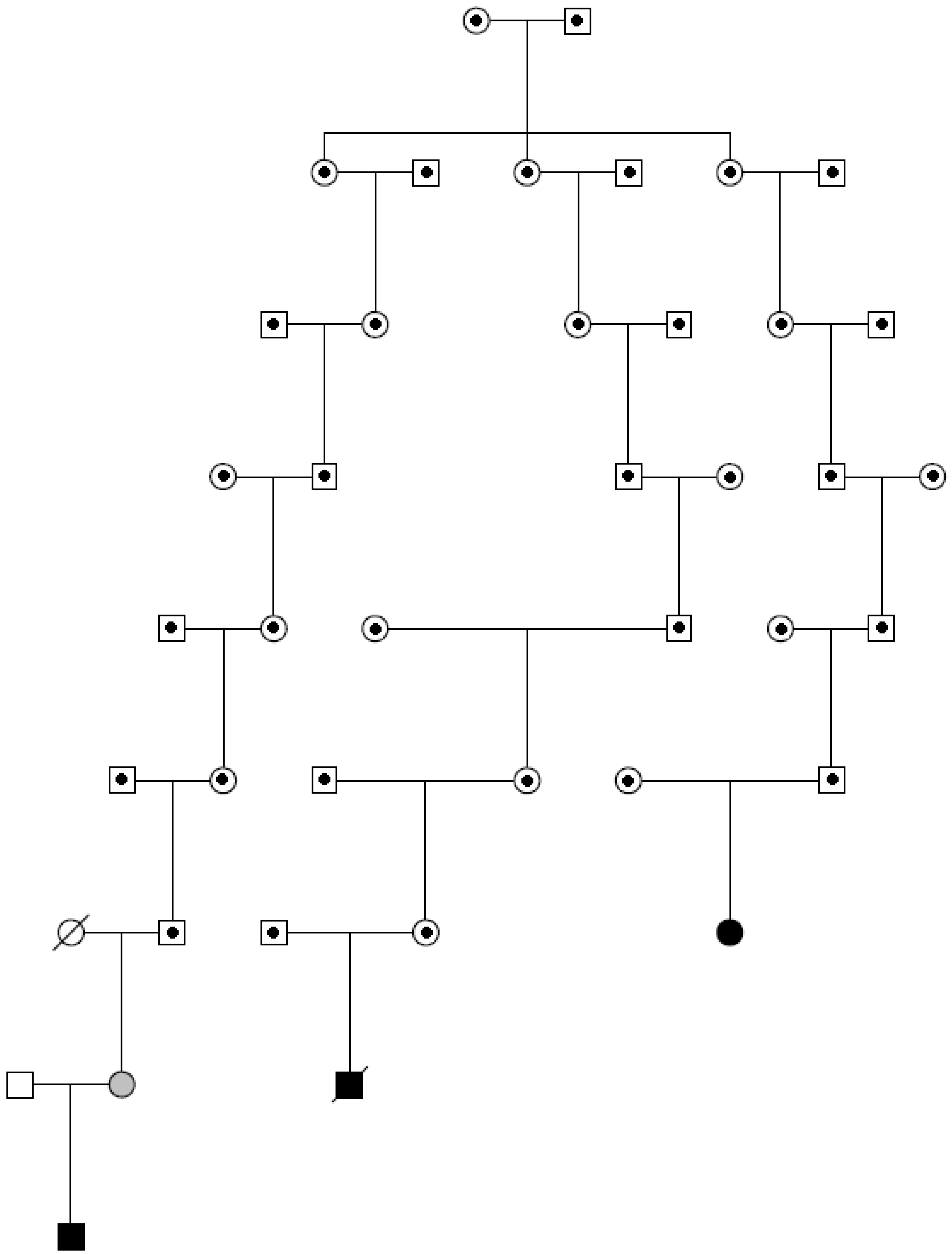

Family 23

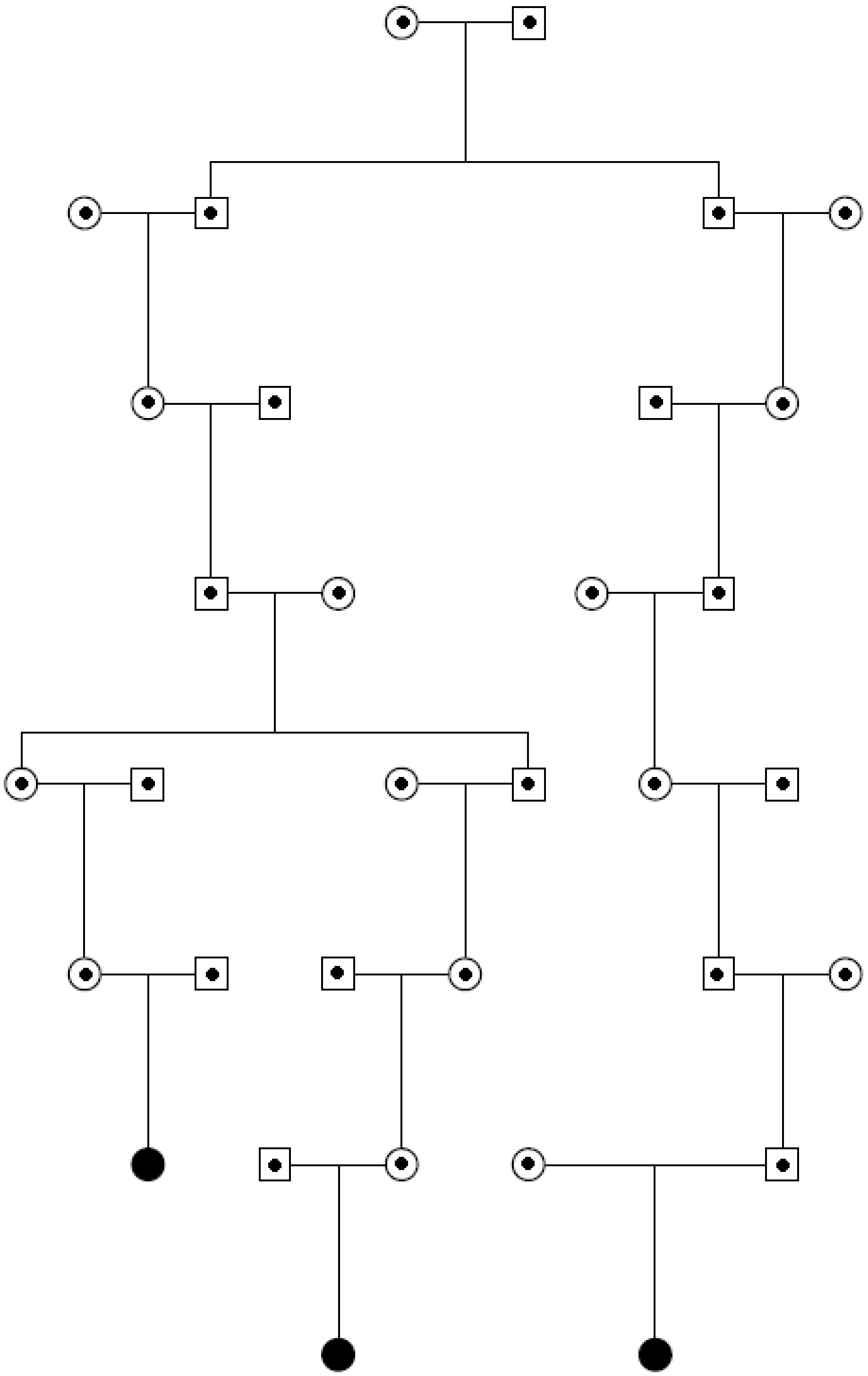

Family 24

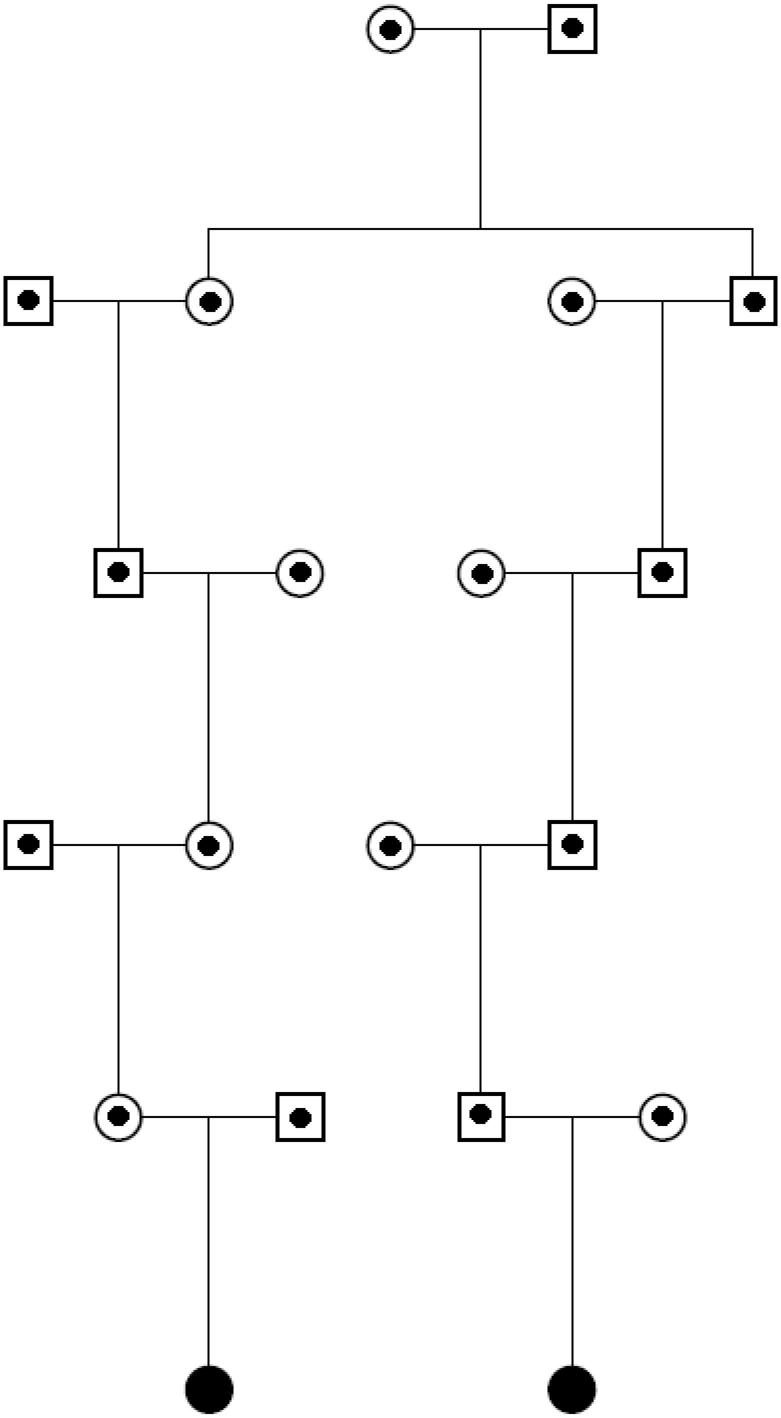

Family 25

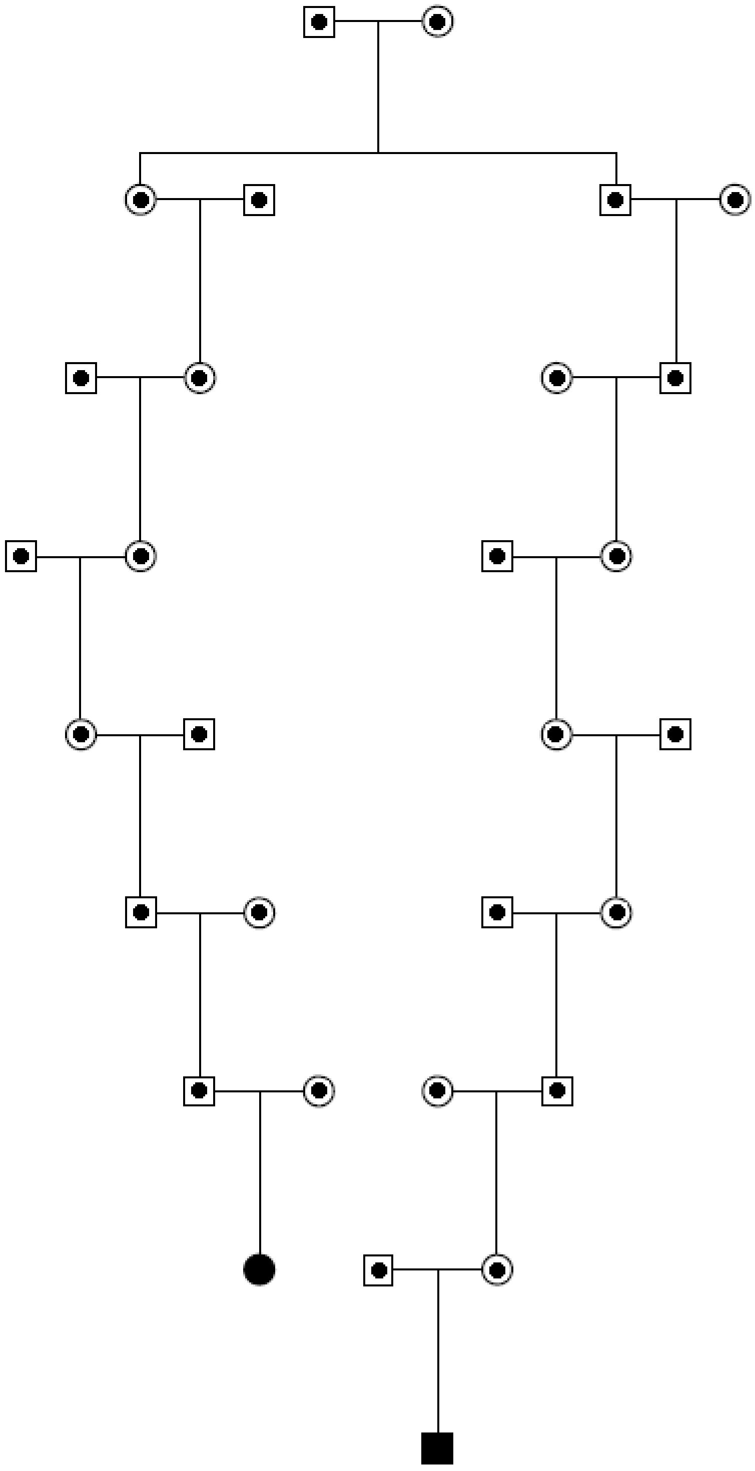

Family 26

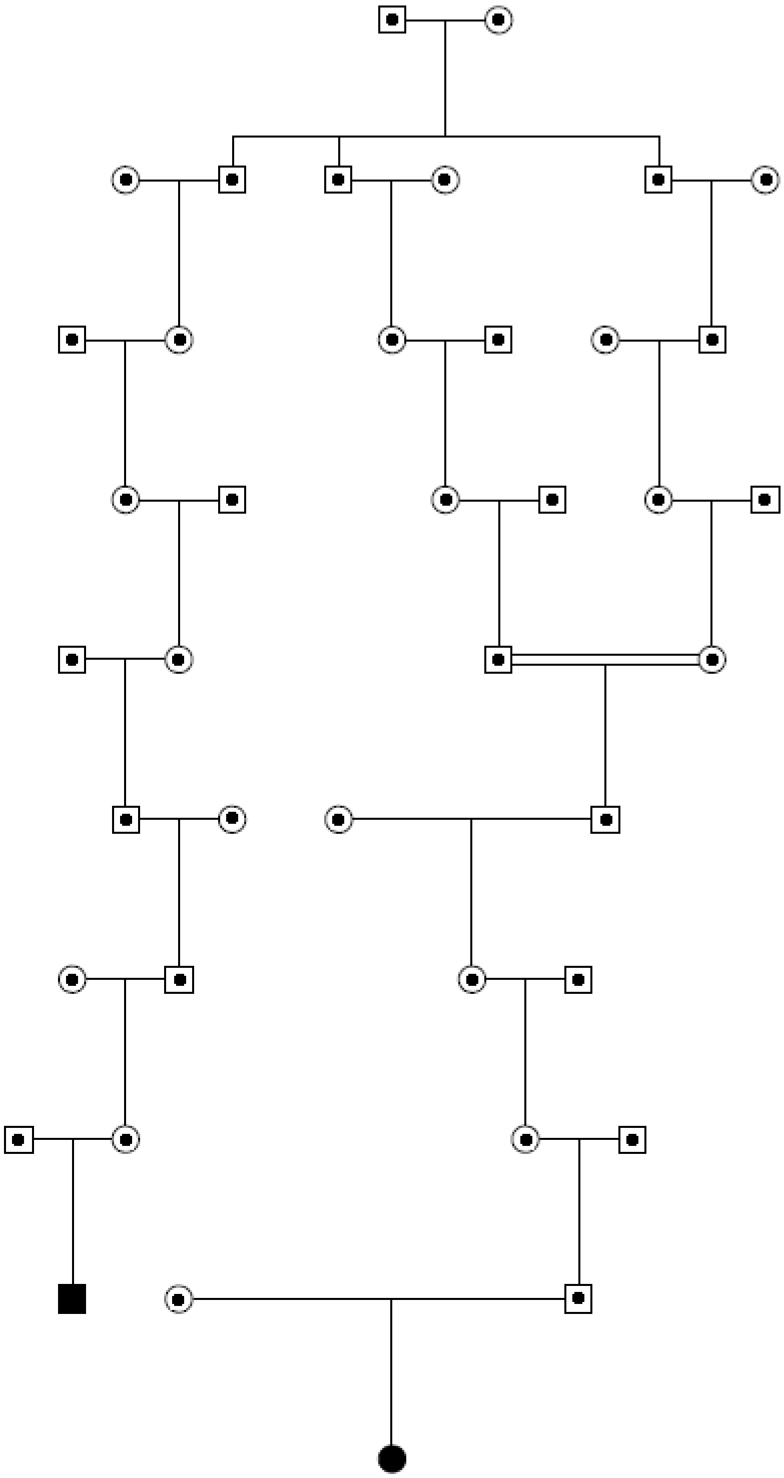

Family 27

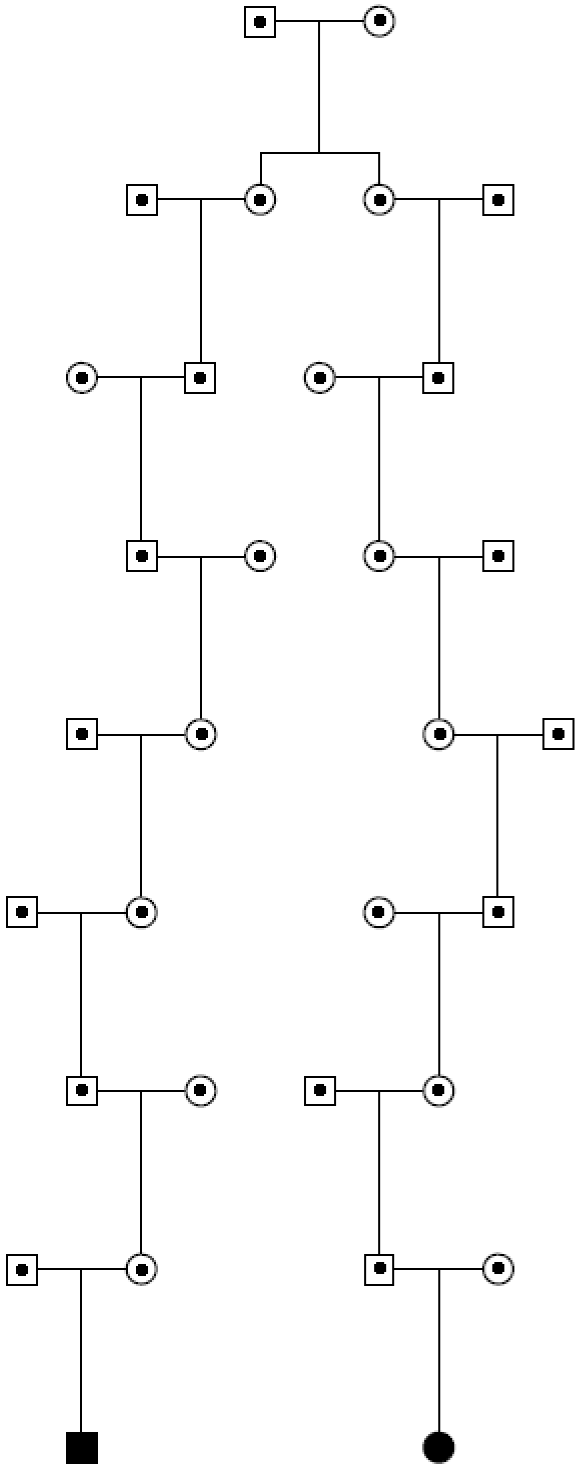

Supplement: Supplementary file 2 [file Image_2.PDF]
